# Morphological, genetic and molecular characteristics of barley root hair mutants

**DOI:** 10.1007/s13353-014-0225-x

**Published:** 2014-06-05

**Authors:** Beata Chmielewska, Agnieszka Janiak, Jagna Karcz, Justyna Guzy-Wrobelska, Brian P. Forster, Malgorzata Nawrot, Anna Rusek, Paulina Smyda, Piotr Kedziorski, Miroslaw Maluszynski, Iwona Szarejko

**Affiliations:** 1 Department of Genetics, University of Silesia, Jagiellonska 28, 40-032 Katowice, Poland; 2 Scanning Electron Microscopy Laboratory, University of Silesia, Jagiellonska 28, 40-032 Katowice, Poland; 3 The James Hutton Institute, Invergowrie, Dundee, DD2 5DA Scotland UK; 4 Present Address: Plant Breeding and Genetics Laboratory, Joint FAO/IAEA Division, IAEA Laboratories, A-2444 Seibersdorf, Vienna, Austria; 5 Present Address: Plant Breeding and Acclimatization Institute-National Research Institute, Mlochow Research Centre, Platanowa 19, 08-831 Mlochow, Poland

**Keywords:** Agrobotanical analysis, Barley, Genetic analysis, Molecular mapping, Mutants, Root hairs

## Abstract

Root hairs are tubular outgrowths of specialized epidermal cells called trichoblasts. They affect anchoring plants in soil, the uptake of water and nutrients and are the sites of the interaction between plants and microorganisms. Nineteen root hair mutants of barley representing different stages of root hair development were subjected to detailed morphological and genetic analyses. Each mutant was monogenic and recessive. An allelism test revealed that nine loci were responsible for the mutated root hair phenotypes in the collection and 1–4 mutated allelic forms were identified at each locus. Genetic relationships between the genes responsible for different stages of root hair formation were established. The linkage groups of four loci *rhl1*, *rhp1*, *rhi1* and *rhs1*, which had previously been mapped on chromosomes 7H, 1H, 6H and 5H, respectively, were enriched with new markers that flank the genes at a distance of 0.16 cM to 4.6 cM. The chromosomal position of three new genes – two that are responsible for the development of short root hairs (*rhs2* and *rhs3*) and the gene that controls an irregular root hair pattern (*rhi2*) – were mapped on chromosomes 6H, 2H and 1H, respectively. A comparative analysis of the agrobotanical parameters between some mutants and their respective parental lines showed that mutations in genes responsible for root hair development had no effect on the agrobotanical performance of plants that were grown under controlled conditions. The presented mutant collection is a valuable tool for further identification of genes controlling root hair development in barley.

**Electronic supplementary material:**

The online version of this article (doi:10.1007/s13353-014-0225-x) contains supplementary material, which is available to authorized users.

## Introduction

A root hair is an extension of a non-dividing epidermal cell known as a trichoblast. A root hair grows *via* the deposition of new membrane and cell wall precursors at the growing tip. Two main steps can be distinguished in root hair morphogenesis – the differentiation (specification) of trichoblasts and root hair development, which can be divided into three stages – root hair initiation, transition to tip growth and root hair elongation. In most angiosperms, mature hair cells (H cells) are shorter than non-hair cells (N cells) along the longitudinal axis of the epidermis of the root. Differences can arise as a result of symmetric divisions followed by an asymmetric expansion (*e.g. Brachypodium distachyon, Hordeum vulgare*), an asymmetric division of a root epidermal cell (*e.g. Oryza sativa*) or by a position-dependent mechanism for trichoblast differentiation (Schiefelbein et al. 2009; Kim and Dolan, 2011; Marzec et al. 2013). The last mechanism is observed in *Arabidopsis thaliana* in which the epidermal cells are organized into files of root hair cells and non-root hair cells. A root hair develops over the junction of two underlying cortical cells, while non-root hair cells lie over a single cortical cell wall (Grierson and Schiefelbein, 2008).

The large collection of root hair mutants and the development of advanced molecular and bioinformatics methods have enabled an understanding of the molecular basis of root hair development in *Arabidopsis*. More than 140 genes responsible for different stages of root hair development have been identified in *Arabidopsis*, many of which have been characterized at the molecular level (Kwasniewski et al. 2013). Despite the different mechanism of trichoblast differentiation, it is likely that root hair development in monocots may be similar to *Arabidopsis*. However, in the case of monocots, there are only a few reports on the isolation of root hair mutants: in barley (*Hordeum vulgare*; Gahoonia et al. 2001; Engvild and Rasmussen, 2004; Kwasniewski and Szarejko, 2006; Janiak and Szarejko, 2007), in maize (*Zea mays*; Wen and Schnable, 1994; Wen et al. 2005; Hochholdinger et al. 2008) and in rice (*Oryza sativa*; Suzuki et al. 2003; Kim et al. 2007; Ding et al. 2009; Yuo et al. 2009; Yuo et al. 2011; Huang et al. 2013a, b). Moreover, only a few genes have been identified at the molecular level (Wen et al. 2005; Kim et al. 2007; Hochholdinger et al. 2008; Yuo et al. 2009; Yuo et al. 2011; Huang et al. 2013a, b).

In this paper we present results of a morphological, structural, genetic and molecular analysis of 19 barley root hair mutants collected in the Department of Genetics of University of Silesia.

## Material

Eighteen root hair mutants representing different stages of root hair development, obtained after the chemical mutagenic treatment of spring barley cultivars: ‘Dema’, ‘Rudzik’, ‘Karat’, ‘Diva’ and ‘Optic’, were analysed. Mutants derived from cvs.: ‘Dema’, ‘Rudzik’, ‘Karat’ and, ‘Diva’ were developed in the Department of Genetics, University of Silesia (Poland), while mutants from cv. ‘Optic’ were selected within the TILLING project at the James Hutton Institute (formerly the Scottish Crop Research Institute, UK). The doses for the treatment of Rudzik and Dema were as follows: 1.5 mM sodium azide (NaN_3_)/3h – inter-incubation germination/6h – 0.75 mM N-methyl-N-nitrosourea (MNU)/3h and for Diva and Karat a double treatment of MNU was used: 0.7 mM MNU/3h – inter-incubation germination/6h – 0.7 mM MNU/3h. Treatments with ethyl methanesulfonate (EMS; 20 mM/16h or 30 mM/16h) were used for Optic (Caldwell et al. 2004). In addition to chemically induced mutants, a spontaneous mutant *brb* (*bald root barley*) from cv. Pallas (Gahoonia et al. 2001), which was kindly provided by the Department of Agricultural Sciences, the Royal Veterinary and Agricultural University, Denmark, was included in the analysis.

Based on observations using a stereoscopic light microscope (LM) and a scanning electron microscope (SEM), the mutants were divided into four phenotypic classes:

- mutants with no root hairs, designated *rhl* (*r* *oot* *h* *air* *l* *ess*): *rhl1.a*, *rhl1.b, rhl1.c* and a spontaneous mutant, *rhl1.d* (*brb*),
- mutants with root hair primordia, which had root hairs arrested at the initial stage of bulge formation or at the very beginning of root hair elongation, designated *rhp* (*r* *oot* *h* *air* *p* *rimordia*): *rhp1.a*, *rhp1.b*, *rhp1.c* and *rhp1.d*,
- mutants with short root hairs, where the elongation of root hairs was stopped, designated *rhs* (*r* *oot* *h* *air* *s* *hort*): *rhs1.a*, *rhs2.a*, *rhs3.a* and *rhs4.a*,
- mutants with sparsely located root hairs of different lengths, designated *rhi* (*r* *oot* *h* *air* *i* *rregular*): *rhi1.a*, *rhi2.a*, *rhi2.b*, *rhi2.c*, *rhi2.d*, or *rhi3.a* and *rhi3.b*.

The mutant names and abbreviations were created after an allelism test, according to the nomenclature recommended for barley (Franckowiak and Lundqvist, 2004).

## Methods

### Growth conditions

Seedlings of the mutants and parental lines were grown in aeroponic conditions. The seeds were sterilized in a 20 % solution of commercial bleach (sodium hypochlorite) for 20 minutes, washed three times in sterile water and then transferred to Petri dishes that were filled with wet vermiculite. The seeds were left at 4 °C overnight and then transferred to a growth chamber at a temperature of 22 °C ± 1 °C for 24 hours in order to start germination. The aeroponic culture was prepared as follows: the germinated seeds were placed in sterile glass tubes with cotton bungs, one seed per tube. Each tube containing a seed was then connected to an empty tube. Both parts were stuck together with parafilm and the bottom tube was wrapped in aluminium foil to protect the roots from light (photos in ESM 1 in Online resources). The plants were grown in the growth room for 5–7 days.

### Light microscopy (LM) and measurements of root hairs

Light microscopic observations and image analysis of 5- to 7-day-old-seedlings were performed using a Stemi 2000-C (Zeiss) stereoscopic microscope and AxioVision LE (Carl Zeiss) software. The length of root hairs was measured for each mutant and its parent. Each experiment consisted of three replications. In each replication, 50 root hairs from a 1 cm segment from the root differentiation zone of five roots (starting from approximately 2.5 cm from the root tip) were measured. Statistical analysis was performed by the analysis of variance (ANOVA) using MSTAT-C package (Michigan State University, USA); significant differences between the mutants and their parents were determined using the LSD test (*P* ≤ 0.05).

### Scanning electron microscopy (SEM)

The 1 cm root segments from the root differentiation zone of 7-day-old seedlings that had been grown in aeroponic culture were excised and immersed in 3 % glutaraldehyde in a 0.1 M sodium phosphate buffer, pH 7.2 for 24 h at room temperature. The roots were then washed three times with the same buffer (15 min each time) and post-fixed in 2 % osmium tetroxide in a phosphate buffer for 2 h at room temperature. Next, the roots were washed in the buffer three times (15 min total) and dehydrated through an ethyl alcohol series (50 %, 60 %, 70 %, 80 %, 90 %, 95 % and 100 %, 10 min at each step). The samples were dried in a Critical Point Pelco-CPD2 apparatus using carbon dioxide and then mounted on aluminium stubs with double-sided tape, sputter coated with gold in a Pelco SC-6 sputter coater and viewed and photographed using a Tesla BS 340 scanning electron microscope at 20 kV. Fomapan Type 400/120 film was used to record the images. At least 50 root segments from each genotype were selected and analysed for the SEM observations.

### Genetic analysis

The inheritance of root hair characters was tested in the F_1_ and F_2_ generations of the crosses among the mutants and between mutants and their parents. Analysis of the F_1_ and F_2_ generations of crosses among the mutants was performed in order to establish the genetic relationships between the mutated genes. In each case, 10–20 F_1_ plants from reciprocal crosses were examined for the root hair phenotype. If F_1_ plants exhibited a mutant phenotype, about 50–200 F_2_ seedlings were checked further in order to confirm the allelic nature of the mutation. When the complementation test was positive, 100–450 F_2_ progeny were analysed in order to establish the genetic interactions between the mutated loci. All of the plants were grown under the aeroponic conditions described above and analysed for root hair phenotype at the 7-day stage. The root hair zone was observed under a stereo microscope and the segregation of root hair characters was tested using the χ^2^_3:1_, χ^2^_9:7_ and or χ^2^_9:3:4_ tests.

### Mapping strategy of the genes affecting root hair morphogenesis

From among the genes that were subjected to the analysis in the presented study, four loci: *rhl1*, *rhp1*, *rhi1* and *rhs1* had previously been mapped in barley chromosomes 7H, 1H, 6H and 5H, respectively (Janiak and Szarejko, 2007). In this study, the linkage groups of these genes were enriched with new markers based on two approaches – Bulked Segregant Analysis (BSA; Michelmore et al. 1991) with AFLP markers and the selection of additional SSR and STS markers from the available genetic maps (Ramsay et al. 2000; Stein et al. 2006; Varshney et al. 2007). Two F_2_ mapping populations, *i.e*. a mutant × ‘Steptoe’ and a mutant × ‘Morex’ were used for each gene for the survey of new markers. The number of F_2_ plants in each mapping population ranged from 199 to 750. For each mapping population, the DNA pools for BSA analysis were composed of DNA samples from 15 individual F_2_ plants with the same root hair phenotype. The markers that were selected from the available genetic maps were first screened in DNA pools. Then, the markers segregating after BSA were analysed using 30 F_2_ plants with the mutant phenotype and those that showed a close linkage to the gene of study were genotyped in the entire F_2_ population, as proposed by Castiglioni and co-workers (1998). The aim of this analysis was to find the markers flanking genes of interest at a distance of less than 1 cM.

Additionally, mapping of two new loci responsible for the development of short root hairs (*rhs2* and *rhs3*) and one gene responsible for irregular root hair distribution (*rhi2*) were initiated. Each mutant was crossed with Steptoe and Morex; thus, two F_2_ mapping populations were created for each mutant. They were analysed using either BSA or the mapping strategy proposed by Castiglioni and co-workers (1998). For the SSR markers that showed a linkage with the trait being analysed, the analysis was extended to all F_2_ plants. To confirm the correct localization of the gene of interest, the AFLP loci of the known position (Hoffman et al. 2000) were examined.

### DNA extraction

Young leaves from individual F_2_ plants, the mutants and Steptoe and Morex were collected in Silica Gel (POCH) and dried for 7 days. After grinding (Retsch MM200 mill), total DNA was extracted using a modified micro-CTAB method (Doyle and Doyle, 1987).

### SSR, STS and AFLP procedures

The PCR reaction for SSR and STS markers was performed in 10 μl of a total reaction volume using 62.5 ng of genomic DNA, 37.5 ng of each primer, 300 μM of each dNTP (Promega), 0.6 U Taq DNA Polymerase (DyNAzyme^TM^II DNA Polymerase) and 1.0 μl of a 1 × PCR buffer (10 mM Tris–HCl, pH 8.8 at 25 °C, 1.5 mM MgCl_2_, 50 mM KCl and 0.1 % Triton X-100). The sequences of SSR primers are given in Table ESM2 (Online resource). PCR conditions for each specific locus were used after Ramsay et al. (2000) and Varshney et al. (2007). The SSR analysis was conducted using non-denaturing or denaturing polyacrylamide gels. In the case of non-denaturing gels, 6 % polyacrylamide gel with no urea (acrylamide/bisacrylamide 19:1 solution, Sigma; 1 × TBE buffer) was used. Electrophoresis was performed under 100 V and gels were later stained in a 5 % solution of ethidium bromide (Sigma) for 10 minutes. If markers were visualized in denaturing gels, a reverse primer labeled with fluorescent dye IRD800 (IBB, MWG) was used in the PCR reaction. Electrophoresis was performed in 6 % polyacrylamide gels (acrylamide/bisacrylamide 19:1 solution, Sigma; 7 M urea, AppliChem; 1 × TBE buffer) in an Li-Cor sequencer (1300 V, 30 mA, 30 W and at a medium speed for laser scanning).

The STS markers that had large size differences between genotypes were directly used for genotyping. Others were subjected to sequencing and screened for SNP polymorphism between the mutants and Steptoe or Morex. SNP polymorphism was then used to develop CAPS markers that were suitable for genotyping. The sequences of STS primers and PCR conditions for their amplification are given in Table ESM3 (Online resource).

The AFLP method was performed according to the Vos et al. (1995) protocol with modifications described in Janiak and Szarejko (2007). The sequences of adaptors and AFLP primers used in the analysis are given in Tables ESM4 and ESM5, respectively (Online resource). Electrophoresis was performed in 6 % denaturing polyacrylamide gels as described for the SSR procedure.

### Data analysis

The linkage analysis was performed using JoinMap 3.0 program (Van Ooijen and Voorrips, 2001). Distances were calculated according to the Kosambi (1944) function and the grouping was estimated using the LOD score of 3.0. Two separate linkage maps were created for each analysed gene – one for the F_2_ mapping population of mutant x Steptoe and the second for mutant x Morex. The maps were then integrated using common markers and the gene of interest from both single maps. Since the linkage groups in this study were compared to the existing maps of Hoffman et al. (2000) and Varshney et al. (2007), the latter two were used as reference maps.

### Agrobotanical analysis of the backcrossed mutants

In order to analyse if mutations affecting the root hair development may have any negative effect on plant performance, when plants are grown in controlled conditions, an analysis of selected agrobotanical characters was carried out. This analysis was performed on four mutants representing four different root hair phenotypes: *rhl1.a* (root hairless), *rhp1.c* (root hair primordia), *rhs2.a* (short root hairs) and *rhi1.a* (root hair irregular). Additionally, three allelic mutants of the *rhi2* locus (*rhi2.a*, *rhi2.b*, *rhi2.c*) were examined. In order to eliminate the probability that other mutations that might exist in the genetic background of a mutant would negatively influence the agrobotanical characters analysed, the mutants were backcrossed twice with their respective parents. For each mutant line, the experiment was performed according to the same scheme – after the first backcross, the F_2_ generation was developed and the individuals that displayed mutant root hairs and that resembled the phenotype of the parental line for other traits were selected and backcrossed to the parent again. Nine seedlings of root hair mutant phenotype were randomly selected from the progeny of one BC_2_F_2_ plant. These recombinants and their respective parents were grown in controlled conditions in a growth chamber (temp. 18–22 °C, light intensity 40 μmol^.^m^−2.^s^−1^ and photoperiod 16h/8h). Seeds were sown individually into pots (13×13×13 cm) filled with commercial soil (Vitahum) mixed with vermiculite (3:1). For each genotype, three replications were set up, where one replication consisted of three pots. Plants were watered every second day and were fertilized with a commercial fertilizer (Florovit, N:P:K=3.0:0.0:2.0) once a week. After harvest, the following measurements were taken: the length of culm and spike, the number of culms bearing spikes, the number of seeds per plant, the weight of seeds per plant and the thousand grains weight (TGW). Statistical analysis was performed using the t-student test using MSTAT-C software (Michigan State University, USA).

## Results

### Root hair phenotypes

**Fig. 1:**
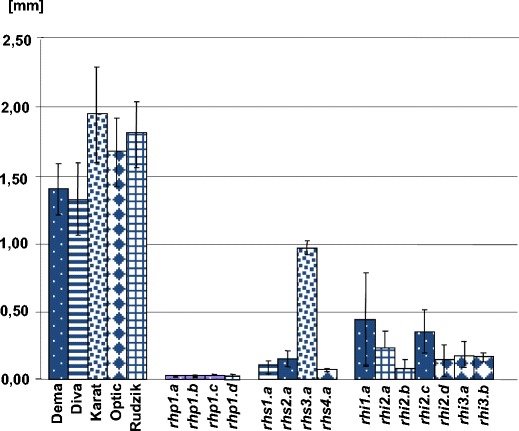
Root hairs length of mutants and their parent lines. In the case of each mutant, there were significant differences in length in comparison to their parent lines

Mutants derived from six parental cultivars – Dema, Diva, Karat, Optic, Pallas and Rudzik were analysed in the presented study. The length of root hairs varied slightly among the parental lines, from 1.31 ± 0.33 mm in Diva to 1.94 ± 0.32 mm in Karat, but the differences were not statistically significant (Fig. 1; ESM6 in Online resource). Observations with light microscopy revealed that the root hairs of the parental lines usually had a regular density, although in some instances fragments with more sparsely located root hairs were present. The observations in SEM proved that the root hair surface zone of all barley cultivars showed a similar arrangement of epidermal cells in a vertical pattern. These epidermal cells were longitudinally elongated with faintly outlined anticlinal cell boundaries. The root hair primordia were arranged at the apical, intermediate or basal site of the epidermal cells with respect to the root apical meristem. The presence of hairs at different developmental stages was fairly common in all of the parent cultivars examined (ESM7 in Online resource). Although most mature root hairs were straight, they were sometimes curled or crooked.

**Fig. 2:**
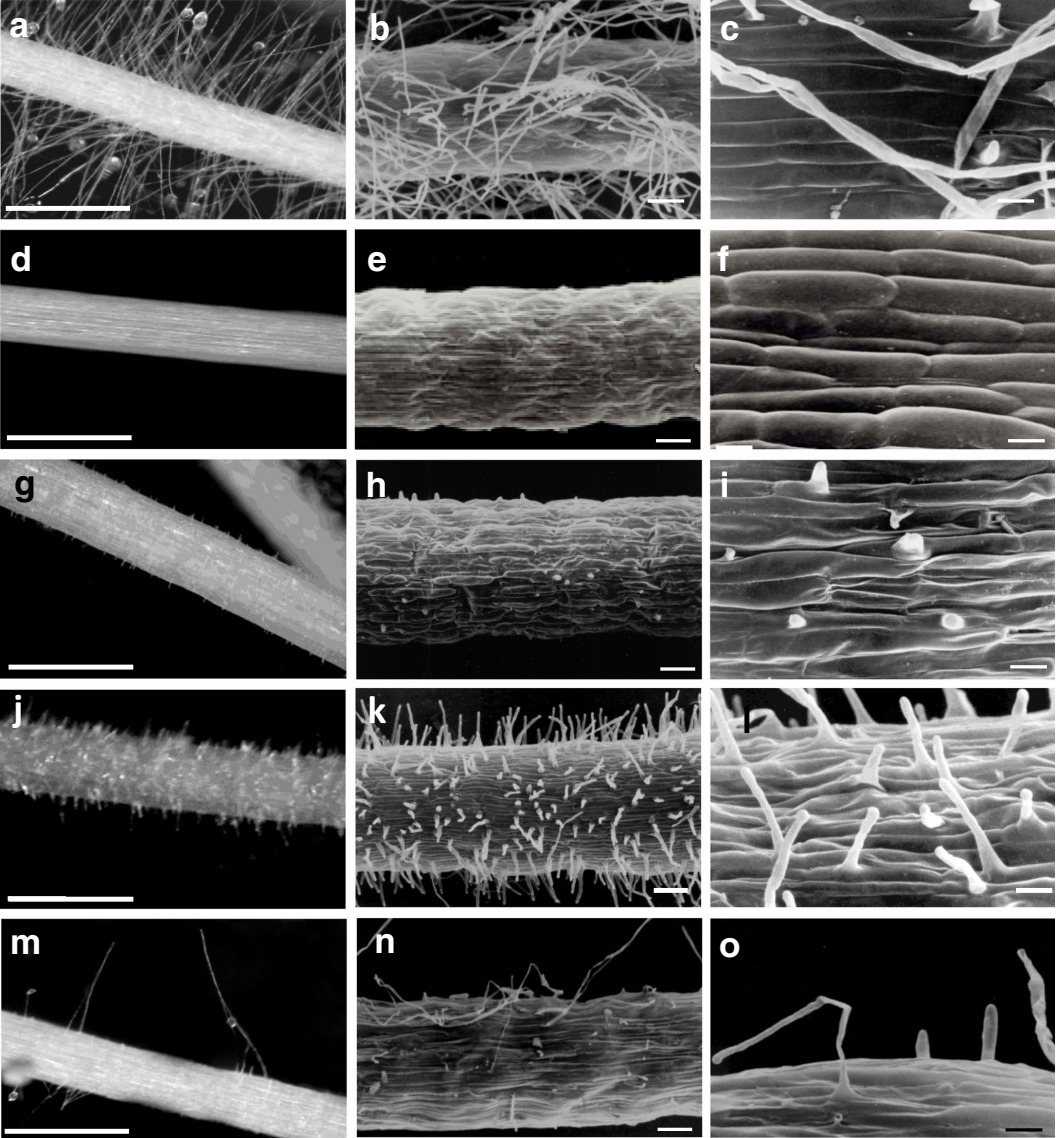
The images of the wild type cultivar and mutants that represent different root hair phenotypes. Root hair surface zone of 5- to 7-d-old root seedlings. The direction of root growth is toward the upper left of each panel. (a,d,g,j,m) LM images of the root hair zone. Bars = 1 mm. (b,c,e,f,h,i,k,l,n,o) SEM images of the root hair zone. (a-c) ‘Dema’, (d-f) *rhl1.a*, (g-i) *rhp1.a*, (j-l) *rhs1.a*, (m-o) *rhi2.c*, scale bar=100 μm in b,e,h,k,n; 20 μm in c,f,i,l,o

All of the mutant lines analysed could be distinguished structurally from the parent lines and displayed the following features: a lack of root hairs, the presence of altered root hairs and/or an abnormal spatial arrangement or distribution pattern of the root hairs (Fig. 2). The root hair morphology was not uniform along the mature root hair surface zone. Similar to the parents, the root hair primordia (emergences) in the mutants were arranged in the same position on the surface of the hair-forming cells.

Detailed SEM observations confirmed that three mutants – *rhl1.a*, *rhl1.b* and *rhl1.c* from Karat and one spontaneous mutant *rhl1.d* from Pallas had not formed root hairs at all (ESM8 in Online resource). All of the epidermal cells were similar in shape and size with clearly visible anticlinal cell walls. This observation indicates that the lack of root hairs in the *rhl* mutants is probably caused by a defect in the differentiation of trichoblasts or by a defect at a very early stage of root hair initiation.

In other mutants, in contrast to the completely root-hairless forms, the root hair morphology was altered. The root hair zone of three mutants – *rhp1.a*, *rhp1.b*, *rhp1.c* from Dema and the *rhp1.d* derived from Rudzik had the same type of phenotype with small root hair primordia that could be observed on the trichoblasts (ESM9 in Online resource). Although the hairs emerged from the epidermal cells, they did not elongate. Occasionally, trichoblasts developed very short hairs with a slightly expanded region at the base of the hair. The length of root hair primordia for all of the *rhp1* mutants was the same (ESM6 and ESM9 in Online resource). It appears that in all of the *rhp* mutants, the trichoblasts formed initiation sites but hair initials failed to undergo the transition to tip growth/elongation.

The root hairs of the mutants *rhs1.a* from Diva, *rhs2.a* from Dema *rhs3.a* from Karat and *rhs4.a* from Optic started to elongate but remained short. Their length ranged from 4.3 % to 49.4 % of the root hair length of the respective parent (Fig. 1; ESM10 in Online resource). The root hairs of the mutant *rhs1.a* (ESM10b, c in Online resource) often exhibited a wavy shape with irregular curvatures along the length of the hair. The root hairs of the mutant *rhs2.a* also exhibited a subtle wavy appearance with slightly wider bases (ESM10e, f in Online resource). The root hairs of the *rhs3.a* mutant had a rather straight shape that was sometimes slightly wavy and were the longest in this phenotypic group (ESM10h, i in Online resource). The root hairs of the *rhs4.a* mutant exhibited a wavy shape that were sometimes curled with wider tips and were the shortest in this phenotypic class (ESM10k, l in Online resource). The *rhs* mutations affected the stage of root hair elongation.

The last group of mutants analysed comprised of seven mutants with irregularly located root hairs of different lengths (ESM11 in Online resource). The variability of root hair length in this class was large – primordia, short and full length root hairs were observed in each mutant (Fig. 1; ESM6 in Online resource). In the case of *rhi1.a* (ESM11a-c in Online resource) and *rhi2.c* (ESM11j-l in Online resource) mutants derived from Dema and *rhi.2a* (ESM11d-f in Online resource) from Rudzik, the distribution of root hairs was not uniform along the root. Almost completely hairless seminal root fragments and roots with a few straight, sometimes curled, very sparsely located root hairs were present on the same plant. The *rhi2.b* mutant derived from Rudzik showed the lowest density of root hairs (ESM11g-i in Online resource). The maximum root hair length of the two mutants derived from Rudzik (*rhi2.a* and *rhi2.b*) was slightly shorter than in the parent (ESM6 in Online resource). The next three mutants *rhi2.d*, *rhi3.a* and *rhi3.b*, which originated from Optic showed the same type of general phenotype – a large portion of the root hair surface zone lacked root hairs and displayed no indication of root hair initiation. A few, irregularly located root hairs of different length were visible on other root fragments (ESM11m-w in Online resource). The maximum root hair length of these three mutants was lower than the medium length of root hairs that was observed in the parent (ESM6 in Online resource).

### Genetic analysis

Crosses between the mutants and their parents revealed that a single recessive gene was responsible for each mutant phenotype. In each case, the F_1_ plants produced wild-type root hairs and the F_2_ progeny segregated at a 3:1 ratio for wild-type to mutant phenotypes. According to the allelism tests, nine loci that control the different stages of root hair development were identified – each locus was represented by 1–4 alleles (Table 1). A single loci, with four alleles each were responsible for the lack of root hairs and also the inhibition of root hair development at the primordium stage, while two other phenotypic groups (short root hairs and irregular root hair pattern) were represented by 4 and 3 loci, respectively, An analysis of the F_2_ generation between the non-allelic mutants revealed that the gene, affecting a lack of root hairs *(rhl1/brb*), was epistatic to all of the other genes that were involved in the further stages of root hair formation, *i.e*. the transition to tip growth (inhibited in *rhp1* mutants), root hair elongation (mutated in *rhs1, rhs2, rhs3* and *rhs4* forms) and the genes that control the distribution of root hair bearing cells (altered in *rhi1, rhi2* and *rhi3* mutants). The 9:4:3 segregation of root hair phenotypes was supported by χ^2^ test in each case (ESM12 in Online resource). An epistatic relationship was also observed between the *rhp1* gene, resulting in the inhibition of root hairs at the primordium stage, and all of the *rhs* genes affecting the length of short root hairs. The value of χ^2^_9:4:3_ test did not exceed the border value of χ^2^_0.05_ = 5.99 for any of the *rhp1* x *rhs* crosses (ESM12 in Online resource).

**Table 1:** Results of alelism test with list of loci

No. of analyzed line	Parent variety	Phenotype	Locus and allele^*^	Number of loci	Number of alleles
834Q	'Karat'	root hairless	*rhl1.a*	1	4
931Q	'Karat'		*rhl1.b*		
934Q	'Karat'		*rhl1.c*		
*brb*	'Pallas'		*rhl1.d*		
DM209.1.3.1	'Dema'	root hair primordia	*rhp1.a*	1	4
DM204.1.8.5	'Dema'		*rhp1.b*		
DM201.11.3	'Dema'		*rhp1.c*		
RD103.1.1	'Rudzik'		*rhp1.d*		
225DV	'Diva'	root hair short	*rhs1.a*	4	1
DM55.1	'Dema'		*rhs2.a*		
740Q	'Karat'		*rhs3.a*		
O03–34	'Optic'		*rhs4.a*		
DM204.1.8.12	'Dema'	root hair irregular	*rhi1.a*	3	1–4
RD100.1	'Rudzik'		*rhi2.a*		
RD101.2.7	'Rudzik'		*rhi2.b*		
DM208.1.6	'Dema'		*rhi2.c*		
O24–85.2	'Optic'		*rhi2.d*		
O08–75	'Optic'		*rhi3.a*		
O19–75.2	'Optic'		*rhi3.b*		

*- In accordance with Franckowiak and Lundqvist, 2004, a number after the gene symbol represents a locus and a letter after the locus number indicates an allele

Similar results were obtained for the crosses between the *rhi1, rhi2 and rhi3* mutants with an irregular root hair pattern and the short root hair *rhs1, rhs2, rhs3* and *rhs4* mutants. The presence of three phenotypic classes in the F_2_ population – plants with normal root hairs, plants with irregular root hairs and plants with short root hairs, segregating at the 9:4:3 ratio suggested that the *rhi* genes, responsible for irregular hair length and distribution, were epistatic over the genes that control root hair elongation. Only in the case of the *rhi1.a* x *rhs1.a* cross did the value of χ^2^_9:4:3_ test higher than the border value, which was caused by the deficit of *rhs1.a* plants (ESM12 in Online resource).

Additionally, the *rhs4* gene was found to be epistatic to other *rhs* genes (ESM13 in Online resource). An analysis of the crosses between two mutants with a similar short root hair morphology, *rhs1.a* and *rhs2.a*, did not permit the mutant phenotypes in the F_2_ progeny of a cross between them to be distinguished; however, epistatic relationships did exist between these two genes (*rhs1* and *rhs2*) and the *rhs3* mutant locus.

The studies of the genetic interactions between the genes permitted the most probable order of their action during root hair morphogenesis in barley to be proposed. The first gene in the pathway is *rhl1*, which is responsible for root-hairless mutant phenotype. The *rhp1* gene, controlling the arrest of root hair transition to tip growth, acts next, followed by the four *rhs* genes, which caused short root hairs. The irregular patterning of the root epidermis is controlled by the *rhi1, rhi2* and *rhi3* loci. They act upstream of the *rhp1* and four *rhs* genes; however, the *rhl* gene product is necessary for the action of the *rhi* genes.

### Mapping of the genes responsible for root hair morphogenesis

In order to map new markers on the four linkage groups that were previously associated with the *rhl1*, *rhp1*, *rhi1* and *rhs1* genes and to find the loci that are most closely linked with the genes, a strategy combining the BSA method and AFLP markers was used. Additionally, other markers were selected from the available genetic maps of barley and were screened in the appropriate mapping population.

**Fig. 3:**
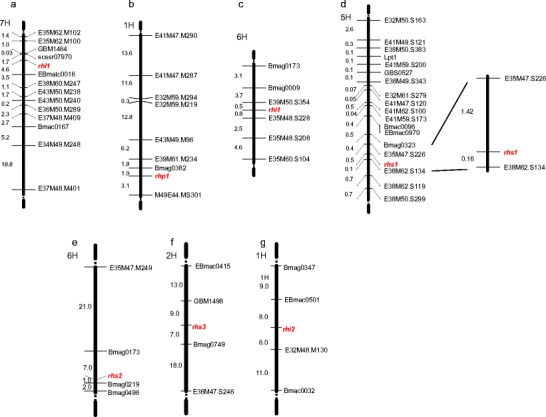
Linkage groups spanning the regions of the genes responsible for root hair development. **a** – Linkage group of *rhl1* gene in chromosome 7H based on the *rhl1.b* x ‘Morex’ mapping population; **b** – Linkage group of *rhp1* gene in chromosome 1H based on the *rhp1.b* x ‘Morex’ mapping population; **c** – Linkage group of *rhi1* gene in chromosome 6H based on the *rhi1.a* x ‘Steptoe’ mapping population; **d** – Linkage group of *rhs1* gene in chromosome 5H based on the *rhs1.a* x ‘Steptoe’ mapping population; on the left: a map constructed using 173 individuals and on the right: the region flanking the *rhs1* gene: map constructed using 750 individuals; **e** – Integrated map for the *rhs2* gene in chromosome 6H; **f** – Integrated map for the *rhs3* gene in chromosome 2H; **g** – Integrated map for the *rhi2* gene in chromosome 1H

All four linkage groups were enriched with new maker loci and the distances between markers and the genes of interest were narrowed down (Table 2). For the *rhl1* gene the strongest linkages were found in the linkage group that was associated with the *rhl1.b* × Morex F_2_ population and two new SSR markers were positioned at a distance of 1.7 cM and 4.6 cM from the gene (Fig. 3a). In the case of the *rhp1* gene only two new markers were possible to map. Current linkage group consists of nine markers and loci flanking *rhp1* gene, the SSR locus Bmag0382, which had been mapped previously, and a new AFLP marker, E44M49.MS301, are located at distances of 1.9 cM and 3.12 cM from the gene, respectively (Fig. 3b). For *rhi1* locus the closest linkages between the gene and the flanking markers were found in the linkage group that was created for *rhi1.a* × Steptoe. One new and one previously mapped AFLP marker were located at distances of 0.5 cM and 0.8 cM from the *rhi1* gene, respectively (Fig. 3c). For the last gene, *rhs1*, the closest linkage to the gene was found for the two AFLP markers, that flanked the locus at the distances of 1.42 cM and 0.16 cM, respectively. Moreover, SSR marker Bmag0323, SNP marker GBS0527 and the *Lpt1* gene were mapped in the linkage group containing the *rhs1* locus, thus giving additional anchoring points to other available barley genetic maps (Fig. 3d). The number and type of AFLP primer combinations used for BSA analysis was given in the Online resources ESM14-ESM17).

**Table 2:** Number of new markers mapped in the *rhl1*, *rhp1*, *rhi1* and *rhs1* gene regions.

Gene	No. of new AFLPs mapped	No. of new SSRs mapped	No. of new STSs mapped	Markers flanking the gene (distance to gene)
*rhl1*	5 (M)	2 (M), 3 (S), 1 (M,S)		scssr07970 (1.7 cM) EBmatc0016 (4.6 cM)
*rhp1*	1 (M), 1 (M,S)			Bmag0382 (1.9 cM)
				E44M49.MS301 (3.12 cM)
*rhi1*	3 (M), 1 (S)			E39M50.S354 (0.5 cM)
				E35M48.S228 (0.8 cM)
*rhs1*	9 (S)	1 (S)	2 (S)	E35M47.S226 and (1.42 cM)
				E38M62.S134 (0.16 cM)

M–markers mapped in mutant × Morex F_2_ population

S–markers mapped in mutant × Steptoe F_2_ population

In addition to the enrichment of the previously created linkage groups, the position of the three new genes that are responsible for root hair morphogenesis was established using the available genetic maps. The *rhs2* gene was mapped in the centromere region of chromosome 6H (Fig. 3e, Table 3). This linkage group contains four markers and spans 31 cM. The order and distances between the loci in the integrated map compared to reference map (Varshney et al. 2007) were slightly changed. No segregation distortion was observed for any of SSR loci analysed, but in the case of the AFLP marker, segregation distortion with a predominance of the mutant class was detected. The second gene, *rhs3*, was localized in the distal region of the long arm of chromosome 2H (Fig. 3f, Table 3). This linkage group contains four markers and spans 47 cM. The order of the loci in the integrated map compared to the reference map (Varshney et al. 2007) was not changed. No segregation distortion was observed for any of the analysed loci. The last gene, *rhi2*, was mapped in the centromere region of chromosome 1H (Fig. 3g, Table 3). The linkage group with the *rhi2* gene contains four markers and spans 34 cM. The order between the loci in the integrated map compared to the reference map (Varshney et al. 2007) was not changed. Segregation distortion was detected for one SSR locus and one AFLP marker. The LOD scores of neighboring pairs of markers in the integrated maps for each analysed gene were high and exceeded the value of five in each case (Table 3).

**Table 3:** Results of mapping the genes responsible for root hair morphogenesis

Gene	Mapping population	Number of F_2_ plants analysed	Number of SSRs analysed	Flanking markers / distance to gene	LOD score range*
*rhs2*	*rhs2.a* x 'Steptoe'	165	7	Bmag0173 / 7 cM; Bmag0219 / 1 cM	5.93–34.95
	*rhs2.a* x 'Morex'	169			
*rhs3*	*rhs3.a* x 'Steptoe'	170	41	Bmag0749 / 7 cM; GBM1498 / 9 cM	5.16–28.35
	*rhs3.a* x 'Morex'	190			
*rhi2*	*rhi2.a* x 'Steptoe'	158	16	EBmac0501 / 8 cM; E32M48.M130 / 6 cM	9.18–31.93
	*rhi2.a* x 'Morex'	145			

*range of LOD scores of neighboring pairs of markers in integrated maps

### Agrobotanical analysis of plants grown in controlled conditions

**Fig. 4:**
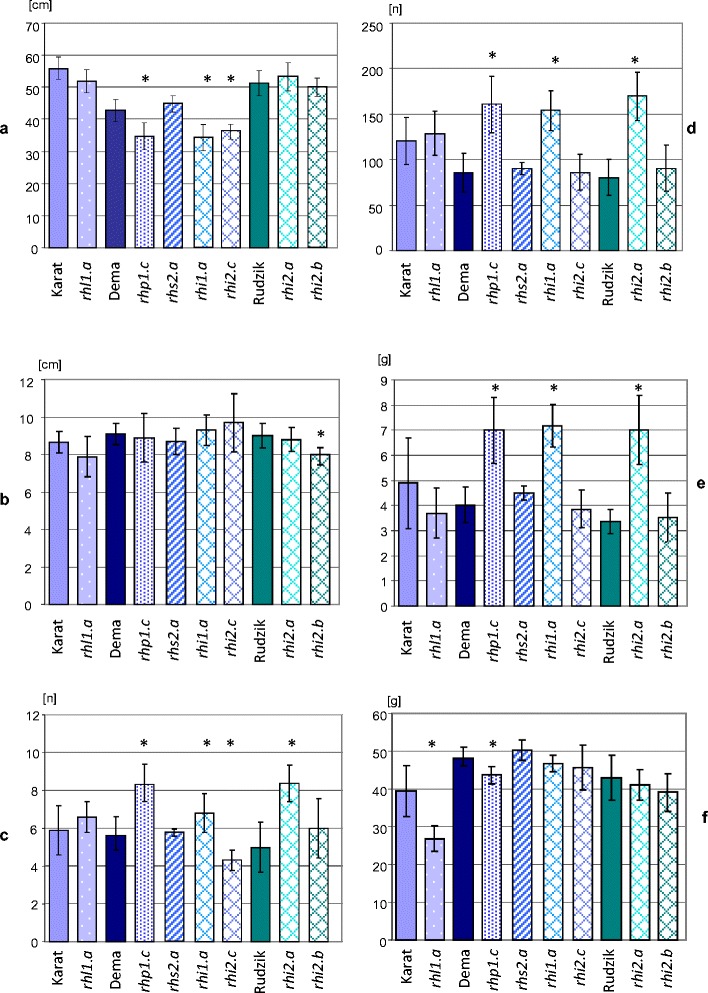
Agrobotanical characters of the mutants after a double backcross and their respective parent lines: **a** – length of culm, **b** – spike, **c** – number of culms bearing spikes, **d** – number of seeds per plant, **e** – weight of seeds per plant, **f** – weight of 1000 seeds. ^*^- an asterisk indicates a significant statistical difference, *P* = 0.05, between the mutant and its parent variety. *rhl – root hairless, rhp – root hair primordia, rhs – root hair short, rhi – root hair irregular*

In the present study, a preliminary analysis of the agrobotanical parameters of mutants and their parental lines grown under controlled conditions was performed. The mutants with altered root hair morphology exhibited higher, the same and only occasionally lower parameters of agrobotanical characters in comparison to their parent lines (Fig. 4). In the case of root-hairless mutants, only the TGW was significantly lower (68 %) than the TGW that was observed in the parent (Fig. 4). In the case of mutants with root hair primordia only (*rhp1.c*) and irregular root hair pattern (*rhi1.a*), the culm length was significantly shorter than that observed in the parent, reaching 81 % and 65 %, respectively. However, the number of culms, number of seeds per plant and the weight of seeds significantly exceeded those of the parent. The mutant *rhi2.a* displayed almost twice the number of culms and number and weight of seeds per plant than the parental line. Two other mutants with sparsely located root hairs (*rhi2.b* and *rhi2.c*), allelic to the *rhi2.a*, displayed yield parameters (number and weight of seeds per plant, TGW) that were similar to their respective parents (Fig. 4). It can be concluded that the morphology of root hairs in the mutants that were studied does not influence the yield in barley grown under optimal and controlled conditions in the growth chamber.

## Discussion

### Root hair phenotypes

A mutation in the *rhl1/brb* gene associated with the root-hairless phenotype that was observed in the four *rhl* allelic forms of mutants analysed in the current study. Marzec et al. (2013) revealed that the epidermal cells in the allelic mutants *rhl1.b* and *brb* were homogeneous with respect to both their length and cytoplasm density. This phenotype suggests that the mutation occurred in the gene that is responsible for controlling the fate of the epidermal cell. To date, there have been no other reports on mutants that are impaired during the differentiation of tricho- and atrichoblasts in monocotyledons.

In the study of the mutants *rhl1.a* and *rhl1.b*, Kwasniewski and Szarejko (2006) showed that there is a lack of the expression of the *HvEXPB1* gene that encodes root specific β-expansin, which affects loosening the cell wall. The sequence of this gene, however, was identical between both the mutants and their parent, which suggests that the mutation is located in some other, upstream acting gene, probably the transcription factor that is involved in the regulation of the *HvEXPB1* gene. MYB and MADS/SQUA transcription factor recognition sites were found in the promoter region of the *HvEXPB1* gene and the genes that encode these elements are the most probable candidates for finding the mutation responsible for the phenotype of the *rhl1.a* and *rhl1.b* mutants.

Additionally, expression profiling of the root-hairless mutant *rhl1.a* and its parent Karat revealed ten genes that are potentially involved in the initiation of root hair morphogenesis in barley. These genes encoded the proteins that are associated with the cell wall and membranes, *e.g.* peroxidases, extensins and xyloglucan endotransglycosylase. The expression level of these genes was strongly reduced in the roots of the root-hairless mutant, whereas in the mutants in which the root hairs were blocked at the primordium stage (*rhp1.b*), it was similar to the wild-type parent (Kwasniewski et al. 2010, 2013).

A mutation in the *rhp1* gene arrests root hair development at the primordium stage while mutations in the *rhs* genes influence the formation of short root hairs. A few mutants with root hair primordia or short root hairs have been found in monocots. In maize, three mutants, *rth1*, *rth2* and *rth3* which exhibit such phenotypes were isolated (Wen and Schnable, 1994) and two genes that are responsible for root hair formation were identified. The first one is the *roothairless3* (*rth3*) gene (acc. no. AY265855), which encodes a putative GPI-anchored COBRA-like protein that is specific to monocots. The maize mutant, *rth3* initiates a normal-looking root hair primordia which fails to elongate (Hochholdinger et al. 2008). The second gene, *roothairless1* (*rth1*; acc. no. AY265854), encodes a homolog of an SEC3-like protein, which is a member of a putative exocyst (Wen et al. 2005). Exocyst tethers exocytotic vesicles prior to their fusion. The maize mutant, *rth1*, is affected in root hair elongation, but exhibits normal root hair initiation. These two genes were candidates for analysis in barley *rhp* and *rhs* mutant lines. The barley homologs of maize *rth3* (acc. no. JF421241.1) and *rth1* (acc. no. AK363680) coding sequences were identified, but no mutations in these sequences were found in *rhp1*, *rhs1*, *rhs2*, *rhs3* and *rhs4* mutants (Chmielewska and Szarejko, unpublished). We cannot exclude, that there is a mutation in the promoter region of these homologs. Another candidate gene from rice, *OsAPY1* (*Apyrase 1*; acc. no. Os07g0682800), was also selected for a similar analysis. It encodes the enzymes that are responsible for the hydrolysis of nucleotide triphosphates (NTPs) and/or diphosphates and controls the level of extracellular ATP, which can act as a signaling molecule (Yuo et al. 2009). The rice mutant develops root hairs that are significantly shorter than the parental line (Yuo et al. 2009). Two barley apyrases were identified and their coding and genomic sequences are known, but no mutation in the genomic sequences in the barley root hair mutants analysed and their parent varieties were found (Marzec, Szarejko, unpublished). In addition to the above-mentioned candidates, there are other possible factors that may explain the phenotypes of the *rhp* and *rhs* mutant lines. The *rhp1* and *rhs* genes may encode proteins that are involved in cell signaling. Foreman et al. (2003) discovered that an *A. thaliana rhd2* mutant, which develops very short root hairs, carries a mutation in the gene encoding NADPH/RHD2 oxidase. This enzyme is involved in the formation of ROS (Reactive Oxygen Species), which are responsible for the activation of the Ca^2+^ channels. Ca^2+^ uptake is one of the factors that stimulates cell exocytosis, cytoskeleton rearrangements and cell expansion in roots (Cramer and Jones, 1996; Carroll et al. 1998). Mutation in the NADPH/RHD2 oxidase gene leads to a lack of ROS species and subsequently to a significant reduction of root hair length. Another *A. thaliana* mutant with short root hairs, *rhd3*, has a defective GTP-binding protein, which is also involved in cell signaling and vesicle transportation, particularly between the endoplasmatic reticulum and Golgi apparatus (Zheng et al. 2004). The *RHD2* gene is epistatic to *RHD3* (both mutant alleles are recessive; Schiefelbein and Somerville 1990). The epistatic relationship between the *rhp1* and *rhs* genes that was observed in the presented study may reflect their role in encoding the proteins that are involved in cell signaling, as was the case of the *rhd2* and *rhd3* mutants in *A. thaliana*. Thus, these two factors will be subjected to further analysis in our barley mutant collection based on the candidate gene approach.

It is possible that *rhp1* and *rhs* genes may encode proteins, like formins that participate in nucleating actin polymerization and elongation, bundling actin filaments, binding and bundling microtubules, and therefore are very important in the organization of microfilaments and microtubules. A short root hair mutant with point mutation in *OsFH1* (*Oryza sativa formin homology1*), which was derived from the *Oryza sativa* spp*. japonica* cultivar ‘Dongjin’, was identified in rice. Mutant root hairs were 85 % shorter than those observed in the wild-type plant. Further analysis revealed that short root hairs were observed only when the roots of mutants were grown submerged in a solution, whereas roots grown in air had root hairs of a normal length. The authors suggested that the *Osfh1* mutant is more sensitive to oxygen depletion or an energy shortage than the wild type. Analyses of expression patterns have shown that *OsFH1* mRNA is detectable in all of the tissues and stages that were analysed, and therefore the authors concluded that *OsFH1* is very important in many developmental processes (Huang et al. 2013a). Another rice mutant with short root hairs, which was derived from the *japonica* cultivar Dongjin, *Ossndp1* (*Oryza sativa Sec14-nodulin domain protein1*), had root hairs that reached only 16 % of the length of their parent. In the case of this mutant not only shortening but also branching of root hairs was observed and the shape and length of the hairs were highly heterogeneous. Expression analysis showed that the mature floral organs contained four to five-fold higher levels of *OsSNDP1* mRNA than other analysed samples. *OsSNDP1* shows a high sequence homology with the *Arabidopsis COW1/AtSFH1* gene that encodes a phosphatidylinositol transfer protein (PITP) (Huang et al. 2013b).

Mutations in *rhi* loci, led to the development of irregular, sparsely located root hairs that were different in length. In *Arabidopsis*, similar phenotypes were observed in the mutants *cpc* (*caprice*) or *cpl3* (*caprice-like MYB3*; Schiefelbein, 2003; Tominaga et al. 2008) and *rhl* (*root hairless*) forms (Sugimoto-Shirasu et al. 2005). Mutated genes that are detected in these forms take part in the specification of root epidermal cells and root hair initiation, respectively. The phenotype of *rhi* mutants implies that *rhi* genes may be involved either in the control of root epidermal cell fate or in root hair initiation. In contrast to *Arabidopsis*, where the fate of rhizodermis cells is controlled by positional mechanisms, a different type of root epidermis pattern exists in barley. According to the study of Marzec et al. (2013), the reduction in root hair density and the irregular distribution of root hairs in the *rhi1.a* mutant was correlated with a drastic decrease in the number of trichoblasts that were formed (10 % of the trichoblast number that was in the wild-type parent), while a similar proportion of trichoblasts as in their parent lines was noted in the *rhi2.d* and *rhi3.a* mutants. These observations imply that the *rhi1* gene may be related to the determination of the root epidermis pattern while the *rhi2* and *rhi3* genes may be involved in a further stage of root hair development.

### Mapping of the genes responsible for root hair morphogenesis

The search for new molecular markers that will enrich the four previously created linkage groups of *rhl1*, *rhp1*, *rhi1* and *rhs1* genes permitted the genetic distances between the genes of study and their flanking markers to be narrowed down. These distances ranged from 0.16 cM to 4.6 cM and are smaller than previously reported (Janiak and Szarejko, 2007). Flanking markers were positioned 0.5 and 0.8 cM from the *rhi1* gene and for *rhs1* locus one marker was located 0.16 cM from the gene. These findings fulfil the assumed 1 cM distance which was set as the goal of the analysis presented in this paper. Larger distances were obtained for the remaining two loci. Nevertheless, all new flanking markers can be used for screening large mapping populations in order to find double recombinants between them and the genes of interest. Such recombinants will be used later for high resolution mapping.

Bulked segregant analysis (BSA) coupled with AFLP markers, has frequently been used for screening for close linkages between loci, but usually requires the analysis of a large number of markers. In the present study, 35 to 100 AFLP primer combinations were screened and from two to nine markers closely linked with the genes of interest were found. Other studies show that the effectiveness of finding new markers with BSA method may vary. One example gives an indication that after screening 1894 AFLP primer combinations only 12 loci were linked to the gene of interest (Kikuchi et al. 2003). In another study, BSA strategy coupled with an analysis of 24 SSRs and 193 AFLP primer combinations allowed to identify four loci closely linked to the gene of interest and two markers co-segregating with the gene (Nissan-Azzouz et al. 2005). These results together with our findings show that the BSA strategy may lead to the development of new markers, but it may not necessarily generate markers that are very closely positioned to the gene of interest. Selection of markers from the available genetic and physical maps seems to be a better strategy, especially in light of the recently published sequence of the barley genome (Mayer et al. 2012). These new sources of data may considerably speed up map-based cloning projects, as was previously shown for other species with a sequenced genome (Jander et al. 2002).

### The influence of root hairs on yield

Root hairs are present in almost all vascular plants. It is thought that they play a basic role in the uptake of nutrients because their presence increases the absorptive surface of the root. In the presented study, it was shown that in plants grown under controlled conditions in the growth chamber, the lack of or changes in the morphology of root hairs did not have a significant influence on agrobotanical parameters that were analysed. The only exception was the *rhs1.a* line with short root hairs, which was characterized by a dwarf plant height, very short spikes, awns and roots (data not shown). However, the *rhs1.a* was not included in this experiment as the backcrosses of the mutant to the parent Diva failed to produce recombinants with mutated root hairs and other traits that would resemble the parent. The most probable reason is the pleiotropic effect of a mutated gene that affected not only the root hair length but also the length of culm, spike, awns and roots (J. Guzy-Wrobelska – personal communication). Similar results were obtained in maize grown in a greenhouse and in field conditions, where no connection between the presence of root hairs and the yield was observed for the mutants *rth2* and *rth3*, whereas mutant *rth1* exhibited a much lower vigour that resulted in a lower yield (Wen and Schnable, 1994). In another experiment in barley, the connection between the phenotype of root hairs and their ability to uptake nutrients, especially phosphorous, was established. Barley cultivars with long root hairs, regardless of the availability of phosphorus, preserved a stable grain yield. In contrast, genotypes with short root hairs produced a lower grain yield in low phosphorus soil, but after fertilization, the grain yield was increased (Gahoonia and Nielsen, 2004). Comparable results were obtained by Brown and coworkers (2012) who used barley mutants from Optic, representing various root hair lengths. It was shown that mutants with long root hairs accumulated significantly more P than those with short or no root hairs. However, the yield of lines with long root hairs did not out-perform the short root hair forms, except for the number of tillers. According to the authors, this implies that the metabolic cost of the development of longer root hairs is higher than the gain achieved from the accumulation of more nutrients (Brown et al. 2012). The results suggested that although root hair length was not important for yield in P-sufficient conditions, the presence of root hairs is necessary for a sustainable yield of barley in P-deficient conditions.

A comparison of some agrobotanical characters between a rice *rth2* (*root hairless2*) mutant and its parent cv. ‘Nipponbare’ showed no significant differences between these two genotypes in a pot experiment. Some parameters that were analysed were lower in the mutant than in the wild-type (*e.g.* the number of spikelets per panicle), some exceeded the parent value (*e.g.* plant height) and some were at the same level (*e.g.* TGW) (Yuo et al. 2011). In the present study, a lack of the influence of mutations in the genes responsible for root hair development on agrobotanical characters could result from the sufficient acquisition of nutrients and water by plants grown under controlled conditions. Thus, it can be concluded that root hairs may not influence the yield of barley when the availability of nutrients is sufficient and plants are grown under controlled conditions in the growth chamber. It has to be mentioned, however, that the performance of root hair mutant lines may be different when grown in the field conditions where they may be subjected to changes of temperature and irregularity of rainfalls.

In our parallel work, we examined the role of root hairs on water uptake in barley using global transcriptome profiling of the leaf and root tissues of *rhl1.a* mutant in comparison to its parent cultivar Karat under drought stress (Kwasniewski et al. 2014, in preparation). The comparison of transcriptional changes resulted in the identification of genes that were differentially affected by drought in both genotypes. The general picture that emerged from their functional analysis led to the conclusion that the lack of root hairs leads to much severe stress in mutant leaves and roots and causes damage to its cellular structures while in the parent variety with normal root hairs genes related to drought tolerance are actively induced. Such observation may confirm the importance of root hairs in plant survival during environmental stress conditions.

## Electronic supplementary material

Below is the link to the electronic supplementary material.

ESM 1 (DOC 239 kb)

ESM 2 (DOC 29 kb)

ESM 3 (PPT 941 kb)

ESM 4 (PDF 1.46 mb)

## Abbreviations

AFLP: Amplified fragment length polymorphism
BSA: Bulked segregant analysis
EMS: Ethyl methane sulphonate
LM: Light microscopy
MNU: N-methyl-N-nitroso urea
NaN_3_: Sodium azide
*rhl*: *root hairless*
*rhp*: *root hair primordia*
*rhs*: *root hair short*
*rhi*: *root hair irregular*
SEM: Scanning electron microscopy
SSR: Simple sequence repeat
TGW: Thousand grain weight
