# Supplementary material for: Morphological, genetic and molecular characteristics of barley root hair mutants
Source: J Appl Genet. 2014 Jun 5;55(4):433–47. doi: 10.1007/s13353-014-0225-x (PMC4185097; doi:10.1007/s13353-014-0225-x)
Supplement: Supplementary file 1 — (DOC 239 kb) [file 13353_2014_225_MOESM1_ESM.doc]

Morphological, genetic and molecular characteristics of barley root 
hair mutants

Journal of Applied Genetics

Beata Chmielewska^1^, Agnieszka Janiak^1^, Jagna Karcz^2^, Justyna Guzy-Wrobelska^1^, Brian P. Forster^3^, Malgorzata Nawrot^1^, Anna Rusek^1^, Paulina Smyda^1^, Piotr Kędziorski^1^, Miroslaw Małuszynski^1^ and Iwona Szarejko^1^

^1^ Department of Genetics, University of Silesia, Jagiellońska 28, 40-032 Katowice, Poland;

^2^ Scanning Electron Microscopy Laboratory, University of Silesia, Jagiellońska 28, 40-032 Katowice, Poland;

^3^ The James Hutton Institute, Invergowrie, Dundee DD2 5DA, Scotland, UK. Current address: Plant Breeding and Genetics Laboratory, Joint FAO/IAEA Division, IAEA Laboratories, A-2444 Seibersdorf, Austria

corresponding author: [iwona.szarejko@us.edu.pl](mailto:iwona.szarejko@us.edu.pl), tel: +48 322009570

**Supplementary tables (ESM tables)**

ESM2. SSR primer sequences used for the amplification of SSR loci

| **Name of SSR marker** | **Sequence 5’ – 3’** | **Quality** | **Mutant** |
| --- | --- | --- | --- |
| Bmag0007 | A: TGAAGGAAGAATAAACAACCAACA | 2 | *rhs3.a* |
|  | B: TCCCCTATTATAGTGACGGTGTG |  |  |
| Bmag0009 | A: AAGTGAAGCAAGCAAACAAACA | 2 | *rhs2.a, rhs3.a* |
|  | B: ATCCTTCCATATTTTGATTAGGCA |  |  |
| Bmag0013 | A: AAGGGGAATCAAAATGGGAG | 3 | *rhs3.a* |
|  | B: TCGAATAGGTCTCCGAAGAAA |  |  |
| Bmag0021 | A: ATTTTTATCAGAACGTCTCTCTC | 2 | *rhs3.a* |
|  | B: CTAACTTCTCTCTCCCTCTCC |  |  |
| Bmag0105 | A: AATCAGACCCATCAGAGGT | 1 | *rhs3.a, rhi2.b* |
|  | B: CCGGTCTCATAGAAATGG |  |  |
| Bmag0120 | A: ATTTCATCCCAAAGGAGAC | 2 | *rhs3.a* |
|  | B: GTCACATAGACAGTTGTCTTCC |  |  |
| Bmag0125 | A: AATTAGCGAGAACAAAATCAC | 2 | *rhs3.a* |
|  | B: AGATAACGATGCACCACC |  |  |
| Bmag0138b | A: ACCAGGAGGAATGAGAGAG | 2 | *rhi2.b* |
|  | B: AATAAACCTTGAGACGATGG |  |  |
| Bmag0173 | A: CATTTTTGTTGGTGACGG | 2 | *rhs2.a, rhs3.a* |
|  | B: ATAATGGCGGGAGAGACA |  |  |
| Bmag0219 | A: ATATTTATGAAACGGTGAAGC | 3 | *rhs2.a* |
|  | B: GGGTTTATCCTCTGGTCC |  |  |
| Bmag0222 | A: ATGCTACTCTGGAGTGGAGTA | 1 | *rhs3.a* |
|  | B: GACCTTCAACTTTGCCTTATA |  |  |
| Bmag0223 | A: TTAGTCACCCTCAACGGT | 2 | *rhs3.a, rhi2.b* |
|  | B: CCCCTAACTGCTGTGATG |  |  |
| Bmag0225 | A: AACACACCAAAAATATTACATCA | 2 | *rhs2.a, rhs3.a, rhi2.b* |
|  | B: CGAGTAGTTCCCATGTGAC |  |  |
| Bmag0323 | A: TTTGTGACATCTCAAGAACAC | 1 | *rhs1.a* |
|  | B: TGACAAACAAATAATCACAGG |  |  |
| Bmag0345 | A: ATGAGGAATAACTCAACCAAA | 1 | *rhs3.a* |
|  | B: AATATATTTTCGATGTCGAGC |  |  |
| Bmag0347 | A: CTGGGATTGGATCACTCTAA | 2 | *rhs3.a, rhi2.b* |
|  | B: AAAACAAGTACTGAAAATAGGAGA |  |  |
| Bmag0353 | A: ACTAGTACCCACTATGCACGA | 1 | *rhs3.a* |
|  | B: ACGTTCATTAAAATCACAACTG |  |  |
| Bmag0378 | A: CTTTTGTTTCCGTAGCATCTA | 2 | *rhs3.a* |
|  | B: ATCCAACTATAGTAGCAAAGCC |  |  |
| Bmag0382 | A: TGAAACCCATAGAGAGTGAGA | 2 | *rhp1.b, rhs3.a, rhi2.b* |
|  | B: TCAAAAGTTTCGTTCCAAATA |  |  |
| Bmag0384 | A: TGTGAGTAGTTCACCATAGACC | 2 | *rhs3.a* |
|  | B: TGCCATTATCATTGTATTGAA |  |  |
| Bmag0496 | A: AGTATAACCAACAGCCGTCTA | 2 | *rhs2.a, rhs3.a, rhi2.b* |
|  | B: CTATAGCACGCCTTTGAGA |  |  |
| Bmag0500 | A: GGGAACTTGCTAATGAAGAG | 1 | *rhs2.a* |
|  | B: AATGTAAGGGAGTGTCCATAG |  |  |
| Bmag0516 | A: ATCTAACCCGAACCTTGAG | 2 | *rhl1.b* |
|  | B: AGCATCCATATATACAATGATACA |  |  |
| Bmag0579 | A: CCTAGATAAGGAACATAGCCA | 1 | *rhs3.a* |
|  | B: CAAAGACCCTAACTCATGTTC |  |  |
| Bmag0603 | A: ATACCATGATACATCACATCG | 1 | *rhs3.a* |
|  | B: GGGGGTATGTACGACTAACTA |  |  |
| Bmag0606 | A: CTATTTGTAATGTATGTATGTCCC | 2 | *rhs3.a* |
|  | B: TCATTGGTCCAGATAATACAA |  |  |
| Bmag0749 | A: CGGATTCTTGAGTAGTCTCTG | 2 | *rhs3.a* |
|  | B: GATCTGTTTTTGTAGAACATGC |  |  |
| Bmac0032 | A: CCATCAAAGTCCGGCTAG | 1 | *rhi2.b* |
|  | B: GTCGGGCCTCATACTGAC |  |  |
| Bmac0040 | A: AGCCCGATCAGATTTACG | 3 | *rhs3.a* |
|  | B: TTCTCCCTTTGGTCCTTG |  |  |
| Bmac0093 | A: CGTTTGGGACGTATCAAT | 2 | *rhs3.a* |
|  | B: GGGAGTCTTGAGCCTACTG |  |  |
| Bmac0134 | A: CCAACTGAGTCGATCTCG | 2 | *rhs3.a, rhi2.b* |
|  | B: CTTCGTTGCTTCTCTACCTT |  |  |
| Bmac0163 | A: TTTCCAACAGAGGGTATTTACG | 1 | *rhs3.a, rhi2.b* |
|  | B: GCAAAGCCCATGATACATACA |  |  |
| Bmac0167 | A: CATTTCCACTTCAAAATATCC | 2 | *rhi2.b* |
|  | B: CCAAAGTTTGAGTGCAGA |  |  |
| Bmac0209 | A: CTAGCAACTTCCCAACCGAC | 1 | *rhs3.a* |
|  | B: ATGCCTGTGTGTGGACCAT |  |  |
| Bmac0213 | A: ATGGATGCAAGACCAAAC | 2 | *rhs3.a, rhi2.b* |
|  | B: CTATGAGAGGTAGAGCAGCC |  |  |
| Bmac0316 | A: ATGGTAGAGGTCCCAACTG | 1 | *rhs3.a, rhi2.b* |
|  | B: ATCACTGCTGTGCCTAGC |  |  |
| EBmac0415 | A: GAAACCCATCATAGCAGC | 2 | *rhs3.a* |
|  | B: AAACAGCAGCAAGAGGAG |  |  |
| EBmac0501 | A: ACTTAAGTGCCATGCAAAG | 2 | *rhs3.a, rhi2.b* |
|  | B: AGGGACAAAAATGGCTAAG |  |  |
| EBmac0541 | A: ACGGATCTACTTTAGCTAGCA | 1 | *rhs3.a* |
|  | B: AAACAACCCCACACAATC |  |  |
| EBmac0602 | A: GATTGGAGCTTCGGATCAC | 1 | *rhs3.a* |
|  | B: CCGTCTAGGGAGAGGTTCTC |  |  |
| EBmac0603 | A: ACCGAAACTAAATGAACTACTTCG | 1 | *rhl1.b, rhs3.a* |
|  | B: TGCAAACTGTGCTATTAAGGG |  |  |
| EBmac0656 | A: TAGACCTCATGCTCATGG | 3 | *rhs3.a, rhi2.b* |
|  | B: GTATGTGTAGGTGTAGGAATGC |  |  |
| EBmac0705 | A: GTGGAAAACTGAGTGAAACTC | 1 | *rhs3.a* |
|  | B: TTGAGGAGAAGTAATGACGAT |  |  |
| EBmac0783 | A: TAGTACGGGGCAGTAGATAGT | 1 | *rhs3.a* |
|  | B: TGCATATGTGTGTACTGCTG |  |  |
| EBmac0871 | A: TGCCTCTGTTGTGTTATTGT | 2 | *rhs3.a* |
|  | B: CCCCAAGTGAACATTGAC |  |  |
| AF022725a | A: AGTATGGGGAATTTATTTGG | 2 | *rhl1.b* |
|  | B: GCTGCAAAGTATGACAATATG |  |  |
| AF043094A | A: CACGGTATAAATATCCACCC | 2 | *rhs3.a, rhi2.b* |
|  | B: ATGGACTCTTCTCCCTGAA |  |  |
| HvLox | A: CAGCATATCCATCTGATCTG | 1 | *rhs3.a* |
|  | B: CACCCTTATTTATTGCCTTAA |  |  |
| HVM11a | A: CCGGTCGGTGCAGAAGAG | 3 | *rhs2.a* |
|  | B: GGAACGAAGGGAGTATTAAGCA |  |  |
| GBM1464 | A: ATAGCCGTGCTCTTGCTCAT | na | *rhl1.b* |
|  | B: CAAGACCACCATTTGCATTG |  |  |
| GBM1498 | A: TGCTCCAACCCAAAAGCTAC | na | *rhs3.a* |
|  | B: GAAGACGACGAGCGGTACTC |  |  |
| scssr07970 | A: TGCATTGGGAGTGCTAGG | na | *rhl1.b* |
|  | B: TGCAAGAAGCCAAGAATACC |  |  |

ESM 3. STS primer sequences and the PCR conditions used for their amplification

| **Name of STS marker** | **Sequence 5’ – 3’** | **PCR conditions** |
| --- | --- | --- |
| Lpt1 | A: GGATACCAAGCACCCAAGAG | Initial denaturation at 94^o^C, 5 min, 8 cycles of: 94^o^C, 1 min, 68^o^C decreasing by 1^o^C in each cycle, 50 s, 72^o^C, 1 min, 27 cycles of: 94^o^C, 50 s, 61^o^C, 50 s, 72^o^C, 50 s; final amplification at 72^o^C, 5 min. |
|  | B: CGTAGGTCAGGCAAGGTTTC |  |
| GBS0527 | A: CACCTCATCACCCTATTTCCAC |  |
|  | B: GGCCTTTTGCAGGGACGTAT |  |

ESM4. AFLP adaptor sequences

| **Name** | **Sequence 5’ – 3’** |
| --- | --- |
| *Eco*RI A1 | CTCGTAGACTGCGTACC |
| *Eco*RI A2 | AATTGGTACGCAGTCTAC |
| *Mse*I A1 | GACGATGAGTCCTGAG |
| *Mse*I A2 | TACTCAGGACTCAT |

ESM 5. AFLP primer sequences

| **Symbol^*^** | Selective  nucleotides | **Sequence 5’ – 3’** |
| --- | --- | --- |
|  | Eco+A | GACTGCGTACCAATTCA |
|  | Mse+C | GATGAGTCCTGAGTAAC |
| E32 | E-AAC | GACTGCGTACCAATTCAAC |
| E35 | E-ACA | GACTGCGTACCAATTCACA |
| E36 | E-ACC | GACTGCGTACCAATTCACC |
| M47 | M-CAA | GATGAGTCCTGAGTAACAA |
| M48 | M-CAC | GATGAGTCCTGAGTAACAC |
| * Symbol of primer according to standard nomenclature of AFLP primers after KeyGene | | |

ESM6. Length of root hairs in short root hair mutants in comparison to the parent variety

| Variety / locus and allele | Average root hairs length | | Average root hairs length in comparison to the parent variety (%) | Range of root hairs length [mm] |
| --- | --- | --- | --- | --- |
|  | [mm] | ± SD |  |  |
| 'Dema' | 1,39 | 0,19 | - | 0,29 - 2,79 |
| 'Diva' | 1,31 | 0,33 | - | 0,27 - 2,71 |
| 'Karat' | 1,94 | 0,32 | - | 0,37 - 3,20 |
| 'Optic' | 1,66 | 0,50 | - | 0,27 - 2,74 |
| 'Rudzik' | 1,80 | 0,31 | - | 0,37 - 3,18 |
| *rhp1.a* | 0,03 | 0,0 | 2,16 | 0,01-0,04 |
| *rhp1.b* | 0,03 | 0,01 | 2,16 | 0,01 - 0,05 |
| *rhp1.c* | 0,03 | 0,01 | 2,16 | 0,01 - 0,07 |
| *rhp1.d* | 0,03 | 0,01 | 1,7 | 0,01 - 0,06 |
| *rhs1.a* | 0,10 | 0,03 | 7,9 | 0,05 - 0,19 |
| *rhs2.a* | 0,15 | 0,06 | 10,8 | 0,03 - 0,20 |
| *rhs3.a* | 0,96 | 0,05 | 49,4 | 0,1 - 1,20 |
| *rhs4.a* | 0,07 | 0,01 | 4,3 | 0,03 - 0,15 |
| *rhi1.a* | 0,44 | 0,34 | 31,6 | 0,01 - 1,41 |
| *rhi2.a* | 0,23 | 0,12 | 12,8 | 0,01 - 1,62 |
| *rhi2.b* | 0,08 | 0,06 | 4,4 | 0,02 - 1,5 |
| *rhi2.c* | 0,34 | 0,16 | 24,5 | 0,03 - 1,32 |
| *rhi2.d* | 0,14 | 0,20 | 8,4 | 0,02 - 1,3 |
| *rhi3.a* | 0,17 | 0,09 | 10,2 | 0,01 - 1,2 |
| *rhi3.b* | 0,16 | 0,03 | 9,6 | 0,01 - 1,3 |

ESM12. Epistatic interactions between the genes responsible for different root hair phenotypes

| Cross  Mutant 1 x Mutant 2 | No. of F_2_ plants | | | | χ^2^ _9:4:3_ |
| --- | --- | --- | --- | --- | --- |
|  | Total | Wild type | Mutant 1 phenotype | Mutant 2 phenotype |  |
| *rhl1.b* x *rhp1.a* | 444 | 265 | 105 | 74 | 2.3 |
| *rhl1.b* x *rhs1.a* | 165 | 83 | 46 | 36 | 2.2 |
| *rhl1.b* x *rhs2.a* | 137 | 84 | 31 | 22 | 1.5 |
| *rhl1.b* x *rhs3.a* | 152 | 93 | 32 | 27 | 1.7 |
| *rhl1.b* x *rhs4.a* | 153 | 87 | 34 | 32 | 0.9 |
| *rhl1.b* x *rhi1.a* | 367 | 203 | 98 | 66 | 0.6 |
| *rhl1.b* x *rhi2.a* | 117 | 77 | 22 | 18 | 4.4 |
| *rhl1.b* x *rhi3.b* | 154 | 80 | 40 | 34 | 1.5 |
| *rhp1.b* x *rhs1.a* | 164 | 94 | 45 | 25 | 1.5 |
| *rhp1.d* x *rhs2.a* | 126 | 82 | 25 | 19 | 3.9 |
| *rhp1.d* x *rhs3.a* | 164 | 96 | 45 | 23 | 2.5 |
| *rhp1.d* x *rhs4.a* | 140 | 90 | 27 | 23 | 3.8 |
| *rhi1.a* x *rhp1.b* | 138 | 84 | 30 | 24 | 1.6 |
| *rhi2.c* x *rhp1.d* | 131 | 71 | 37 | 23 | 0.8 |
| *rhi3.a* x *rhp1.c* | 153 | 92 | 36 | 25 | 1.0 |
| *rhi1.a* x *rhs1.a* | 152 | 88 | 47 | 17 | 6.8 |
| *rhi1.a x rhs2.a* | 114 | 57 | 37 | 20 | 3.4 |
| *rhi1.a x rhs3.a* | 162 | 97 | 42 | 23 | 2.2 |
| *rhi1.a x rhs4.a* | 314 | 182 | 79 | 53 | 0.7 |
| *rhi2.a x rhs1.a* | 138 | 77 | 39 | 22 | 1.2 |
| *rhi2.b x rhs2.a* | 139 | 76 | 45 | 18 | 5.6 |
| *rhi2.a x rhs3.a* | 173 | 103 | 45 | 25 | 2.1 |
| *rhi2.b x rhs4.a* | 320 | 189 | 67 | 64 | 4.4 |
| *rhi3.a x rhs1.a* | 139 | 81 | 34 | 24 | 0.3 |
| *rhi3.b x rhs2.a* | 156 | 97 | 33 | 25 | 2.7 |
| *rhi3.b x rhs3.a* | 159 | 96 | 40 | 23 | 2.0 |
| *rhi3.b x rhs4.a* | 170 | 88 | 42 | 40 | 2.7 |

ESM13. Epistatic interactions between the genes responsible short root hairs

| Cross  Mutant 1 x Mutant 2 | No. of F_2_ plants | | | | χ^2^ _9:4:3_ |
| --- | --- | --- | --- | --- | --- |
|  | Total | Wild type | Mutant 1 phenotype | Mutant 2 phenotype |  |
| *rhs1.a* x *rhs3.a* | 138 | 90 | 31 | 17 | 5.4 |
| *rhs2.a* x *rhs3.a* | 144 | 87 | 29 | 28 | 1.8 |
| *rhs4.a* x *rhs3.a* | 156 | 87 | 40 | 29 | 0.03 |
| *rhs4.a* x *rhs2.a* | 160 | 100 | 31 | 29 | 3.2 |
| *rhs4.a* x *rhs1.a* | 150 | 77 | 41 | 32 | 1.5 |

ESM14. AFLP primer combinations used for the development of new markers linked with the *rhl1* gene.

|  | E32 | E34 | E35 | E36 | E37 | E38 | E40 |
| --- | --- | --- | --- | --- | --- | --- | --- |
| M47 | X |  |  |  |  |  | X |
| M48 |  |  |  | X | X |  | X |
| M49 |  | X |  | X | X |  | X |
| M50 |  | X |  | X | X | X |  |
| M51 | X |  | X | X | X | X |  |
| M55 | X |  | X | X | X | X |  |
| M59 | X |  | X | X | X |  |  |
| M60 |  |  |  | X | X | X |  |
| M61 |  |  |  | X | X |  |  |
| M62 | X |  | X | X |  |  |  |

ESM15. AFLP primer combinations used for the development of new markers linked with the *rhp1* gene.

|  | E32 | E33 | E34 | E35 | E36 | E37 | E38 | E39 | E40 | E43 | E44 | E45 | E46 |
| --- | --- | --- | --- | --- | --- | --- | --- | --- | --- | --- | --- | --- | --- |
| M47 |  |  | X |  |  |  |  | X | X | X | X | X |  |
| M48 |  | X | X |  | X | X |  | X | X | X |  | X |  |
| M49 |  | X | X |  | X | X |  | X | X | X | X | X |  |
| M50 |  | X | X |  | X | X | X | X | X | X | X | X | X |
| M51 | X | X | X | X | X | X | X | X | X | X | X | X | X |
| M55 | X | X | X | X | X | X | X | X | X | X | X | X | X |
| M59 |  | X | X | X | X | X |  | X | X | X |  | X |  |
| M60 |  | X | X | X | X | X | X | X | X | X | X | X | X |
| M61 |  | X | X | X | X | X |  | X | X | X | X | X |  |
| M62 |  | X | X | X | X | X |  | X | X | X |  | X |  |

ESM16. AFLP primer combinations used for the development of new markers linked with the *rhi1* gene.

|  | E32 | E33 | E34 | E35 | E36 | E37 | E38 | E39 | E40 | E43 | E44 | E45 | E46 |
| --- | --- | --- | --- | --- | --- | --- | --- | --- | --- | --- | --- | --- | --- |
| M47 |  | X |  |  |  |  |  |  |  |  |  |  |  |
| M49 |  | X | X |  | X | X |  | X | X | X | X | X |  |
| M50 | X | X | X |  | X | X | X | X | X | X | X |  | X |
| M51 |  | X | X |  |  | X |  |  |  | X |  | X | X |
| M55 |  |  |  |  |  |  |  |  |  |  |  |  |  |
| M59 | X | X | X | X | X | X |  | X | X | X |  | X |  |
| M60 | X | X | X |  | X | X | X | X | X | X | X | X | X |
| M61 |  |  |  |  |  |  |  |  | X |  |  | X |  |
| M62 | X | X |  |  |  |  |  | X | X | X |  | X |  |

ESM17. AFLP primer combinations used for the development of new markers linked with the *rhs1* gene.

|  | E32 | E33 | E34 | E35 | E36 | E37 | E38 | E39 | E40 | E41 | E43 | E44 | E45 |
| --- | --- | --- | --- | --- | --- | --- | --- | --- | --- | --- | --- | --- | --- |
| M47 | X |  |  |  | X |  |  |  |  |  |  |  |  |
| M48 |  | X | X | X | X | X | X | X | X | X | X | X | X |
| M49 |  | X | X | X | X | X | X | X | X | X | X | X | X |
| M50 |  | X | X | X | X | X | X | X | X | X | X | X | X |
| M51 | X | X | X | X |  | X | X | X | X | X | X | X | X |
| M55 | X | X | X |  |  | X | X | X | X | X | X | X | X |
| M59 |  | X | X | X | X | X | X | X | X | X | X | X | X |
| M60 |  | X | X | X | X | X | X | X | X | X | X | X | X |
| M61 |  | X |  | X |  |  |  |  |  |  |  |  |  |
| M62 |  | X | X | X | X | X | X | X | X | X | X | X | X |
