# Supplementary material for: Morphological, genetic and molecular characteristics of barley root hair mutants
Source: J Appl Genet. 2014 Jun 5;55(4):433–47. doi: 10.1007/s13353-014-0225-x (PMC4185097; doi:10.1007/s13353-014-0225-x)
Supplement: Supplementary file 2 — (DOC 29 kb) [file 13353_2014_225_MOESM2_ESM.doc]

Morphological, genetic and molecular characteristics of barley root 
hair mutants

Journal of Applied Genetics

Beata Chmielewska^1^, Agnieszka Janiak^1^, Jagna Karcz^2^, Justyna Guzy-Wrobelska^1^, Brian P. Forster^3^, Malgorzata Nawrot^1^, Anna Rusek^1^, Paulina Smyda^1^, Piotr Kędziorski^1^, Miroslaw Małuszynski^1^ and Iwona Szarejko^1^

^1^ Department of Genetics, University of Silesia, Jagiellońska 28, 40-032 Katowice, Poland;

^2^ Scanning Electron Microscopy Laboratory, University of Silesia, Jagiellońska 28, 40-032 Katowice, Poland;

^3^ The James Hutton Institute, Invergowrie, Dundee DD2 5DA, Scotland, UK. Current address: Plant Breeding and Genetics Laboratory, Joint FAO/IAEA Division, IAEA Laboratories, A-2444 Seibersdorf, Austria

corresponding author: [iwona.szarejko@us.edu.pl](mailto:iwona.szarejko@us.edu.pl), tel: +48 322009570

**Legends of Supplementary figures (ESM figures)**

ESM1. Aeroponic culture. (a) germinated seeds on Petri dish, (b) glass tube with cotton bung, (c) a germinated seed placed in sterile tube (embryo directed down), (d) tubes with seeds stuck together with parafilm, (e) bottom tubes wrapped in aluminum foil.

ESM7. Root hair phenotypes of barley cultivars. Root hair surface zone of 5- to 7-d-old root seedlings. The direction of root growth is toward the upper left of each panel. (a,d,g,j,m,p) LM images of the root hair zone. Bars = 1 mm. (b,c,e,f,h,i,k,l,n,o,r,s) SEM images of the root hair zone. (a-c) 'Karat', (d-f) 'Dema', (g-i) 'Diva', (j-l) 'Optic', (m-o) 'Pallas', (p-s) 'Rudzik'; scale bar=100 µm in b,e,h,k,n,r; 20 µm in c,f,i,l,o,s.

ESM8. Root hair phenotypes of barley hairless mutants. Root hair surface zone of 5- to 7-d-old root seedlings. The direction of root growth is toward the upper left of each panel. (a,d,g,j) LM images of the root hair zone. Bar = 1 mm. (b,c,e,f,h,i,k,l) SEM images of the root hair zone showing well the visible epidermal cells without hairs. (a-c) *rhl1.a*, (d-f) *rhl1.b*, (g-i) *rhl1.c*, (j-l) *rhl1.d*; scale bar=100 µm in b,e,h,k; 20 µm in c,f,i,l.

ESM9. Root hair phenotypes of barley root hair primordia mutants. Root hair surface zone of 5- to 7-d-old root seedlings. The direction of root growth is toward the upper left of each panel. (a,d,g,j) LM images of the root hair zone. Bar = 1 mm. (b,c,e,f,h,i,k,l) SEM images of the root hair zone showing hair initiation on the trichoblast cells. (a-c) *rhp1.a*, (d-f) *rhp1.b*, (g-i) *rhp1.c*, (j-l) *rhp1.d*; scale bar=100 µm in b,e,h,k; 20 µm in c,f,i,l.

ESM10. Root hair phenotypes of barley short root hairs mutants. Root hair surface zone of 5- to 7-d-old root seedlings. The direction of root growth is toward the upper left of each panel. (a,d,g,j) LM images of the root hair zone. Bars = 1 mm. (b,c,e,f,h,i,k,l) SEM images of the root hair zone with shorter hairs compared to the parental line. (a-c) *rhs1.a*, (d-f) *rhs2.a*, (g-i) *rhs3.a*; (j-l) *rhs4.a*; scale bar=100 µm in b,e,h,k; 20 µm in c,f,i,l.

ESM11. Root hair phenotypes of barley irregular root hair mutants. Root hair surface zone of 5- to 7-d-old root seedlings. The direction of root growth is toward the upper left of each panel. (a,d,g,j,m,p,t) LM images of the root hair zone. Bars = 1mm. (b,c,e,f,h,i,k,l,n,o,r,s,u,w) SEM images of the root hair zone with a few hairs sparsely located on the epidermal cells. (a-c) *rhi1.a*, (d-f) *rhi2.c*, (g-i) *rhi2.a*, (j-l) *rhi2.b*, (m-o) *rhi2.d*, (p-s) *rhi3.a*, (t-w) *rhi3.b*; scale bar=100 µm in b,e,h,k,n,r,u; 20 µm in c,f,i,l,o,s,w.
