# Supplementary material for: Morphological, genetic and molecular characteristics of barley root hair mutants
Source: J Appl Genet. 2014 Jun 5;55(4):433–47. doi: 10.1007/s13353-014-0225-x (PMC4185097; doi:10.1007/s13353-014-0225-x)
Supplement: Supplementary file 3 — (PPT 941 kb) [file 13353_2014_225_MOESM3_ESM.ppt]

## Slide 1
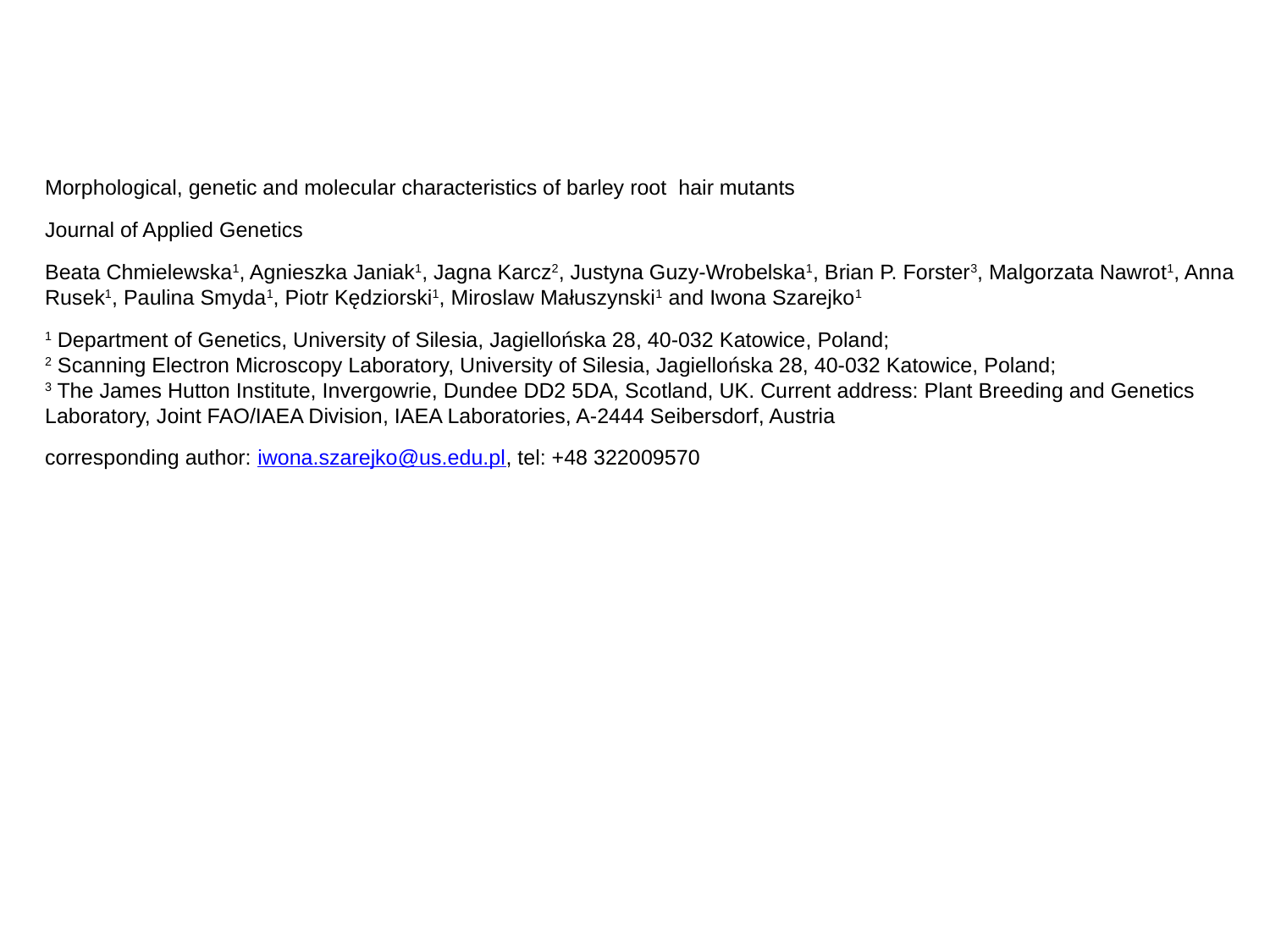

Morphological, genetic and molecular characteristics of barley root  hair mutants
Journal of Applied Genetics
Beata Chmielewska1, Agnieszka Janiak1, Jagna Karcz2, Justyna Guzy-Wrobelska1, Brian P. Forster3, Malgorzata Nawrot1, Anna Rusek1, Paulina Smyda1, Piotr Kędziorski1, Miroslaw Małuszynski1 and Iwona Szarejko1
1 Department of Genetics, University of Silesia, Jagiellońska 28, 40-032 Katowice, Poland;
2 Scanning Electron Microscopy Laboratory, University of Silesia, Jagiellońska 28, 40-032 Katowice, Poland;
3 The James Hutton Institute, Invergowrie, Dundee DD2 5DA, Scotland, UK. Current address: Plant Breeding and Genetics Laboratory, Joint FAO/IAEA Division, IAEA Laboratories, A-2444 Seibersdorf, Austria
corresponding author: iwona.szarejko@us.edu.pl, tel: +48 322009570

## Slide 2
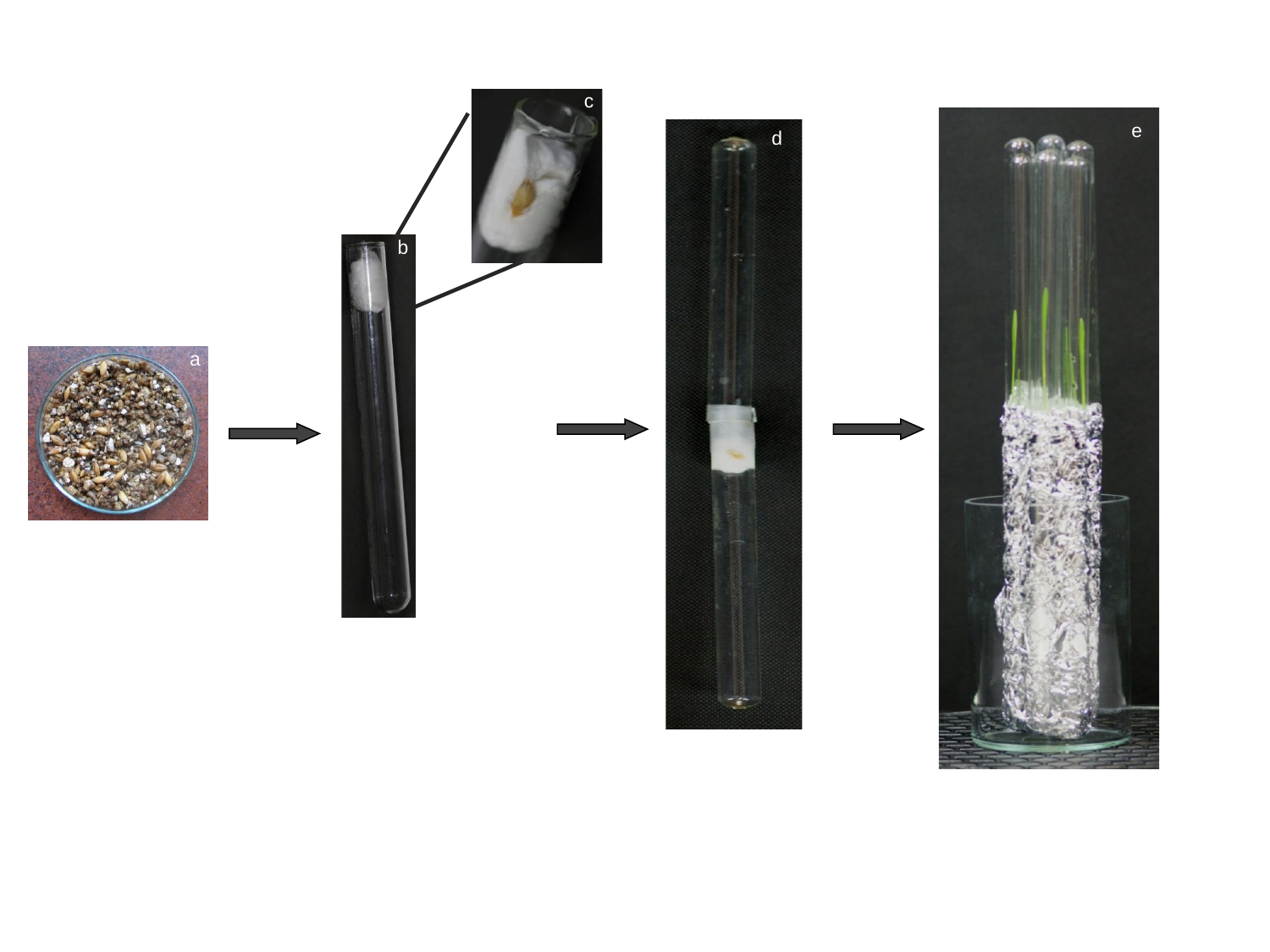

c
e
d
b
a
