# Supplementary material for: Morphological, genetic and molecular characteristics of barley root hair mutants
Source: J Appl Genet. 2014 Jun 5;55(4):433–47. doi: 10.1007/s13353-014-0225-x (PMC4185097; doi:10.1007/s13353-014-0225-x)
Supplement: Supplementary file 4 — (PDF 1.46 mb) [file 13353_2014_225_MOESM4_ESM.pdf]

Morphological, genetic and molecular characteristics of barley root hair mutants

Journal of Applied Genetics

Beata Chmielewska[1], Agnieszka Janiak[1], Jagna Karcz[2], Justyna Guzy-Wrobelska[1], Brian P. Forster[3], Malgorzata Nawrot[1], Anna Rusek[1], Paulina Smyda[1], Piotr Kędziorski[1], Miroslaw Małuszynski[1] and Iwona Szarejko[1]

[1] Department of Genetics, University of Silesia, Jagiellońska 28, 40-032 Katowice, Poland;
[2] Scanning Electron Microscopy Laboratory, University of Silesia, Jagiellońska 28, 40-032 Katowice, Poland;
[3] The James Hutton Institute, Invergowrie, Dundee DD2 5DA, Scotland, UK. Current address: Plant Breeding and Genetics Laboratory, Joint FAO/IAEA Division, IAEA Laboratories, A-2444 Seibersdorf, Austria

corresponding author: iwona.szarejko@us.edu.pl, tel: +48 322009570

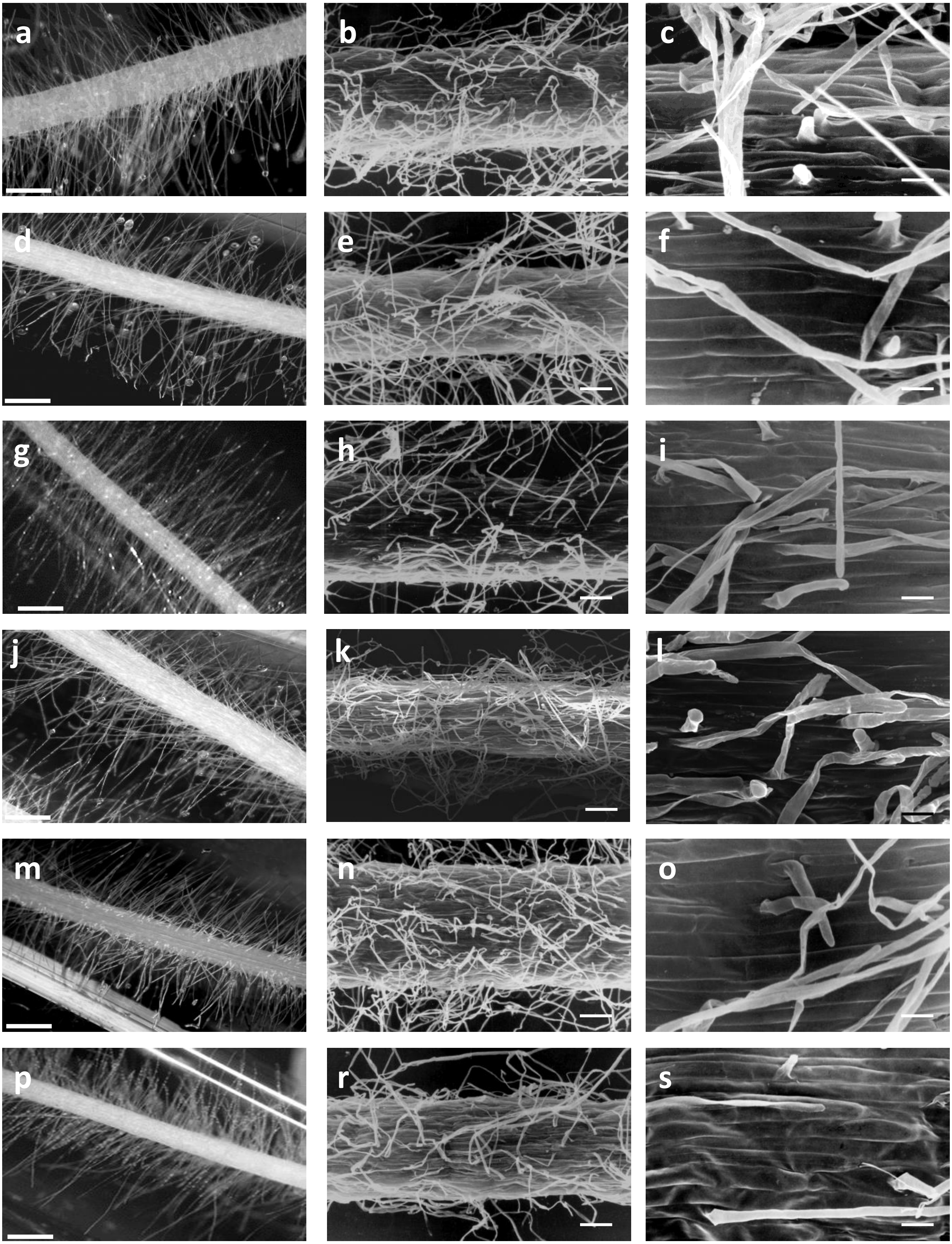

ESM7

a,b,c – Karat; d,e,f – Dema; g,h,i – Diva; j,k,l – Optic; m,n,o – Pallas; p,r,s - Rudzik

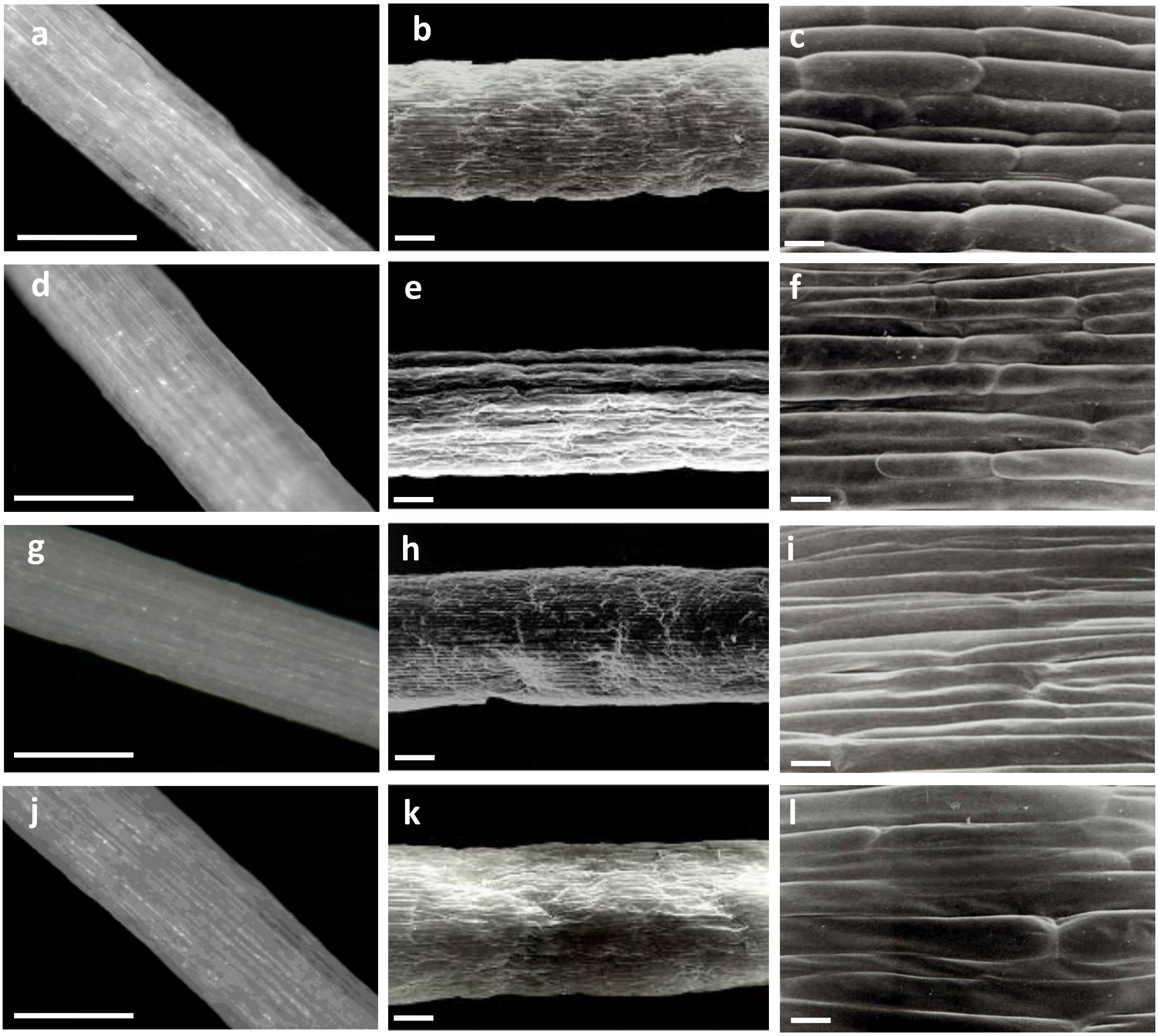

ESM8

a,b,c -*rhl1.a;* d,e,f -*rhl1.b;* g,h,i - *rhl1.c;* j,k,l - *rhl1.d*

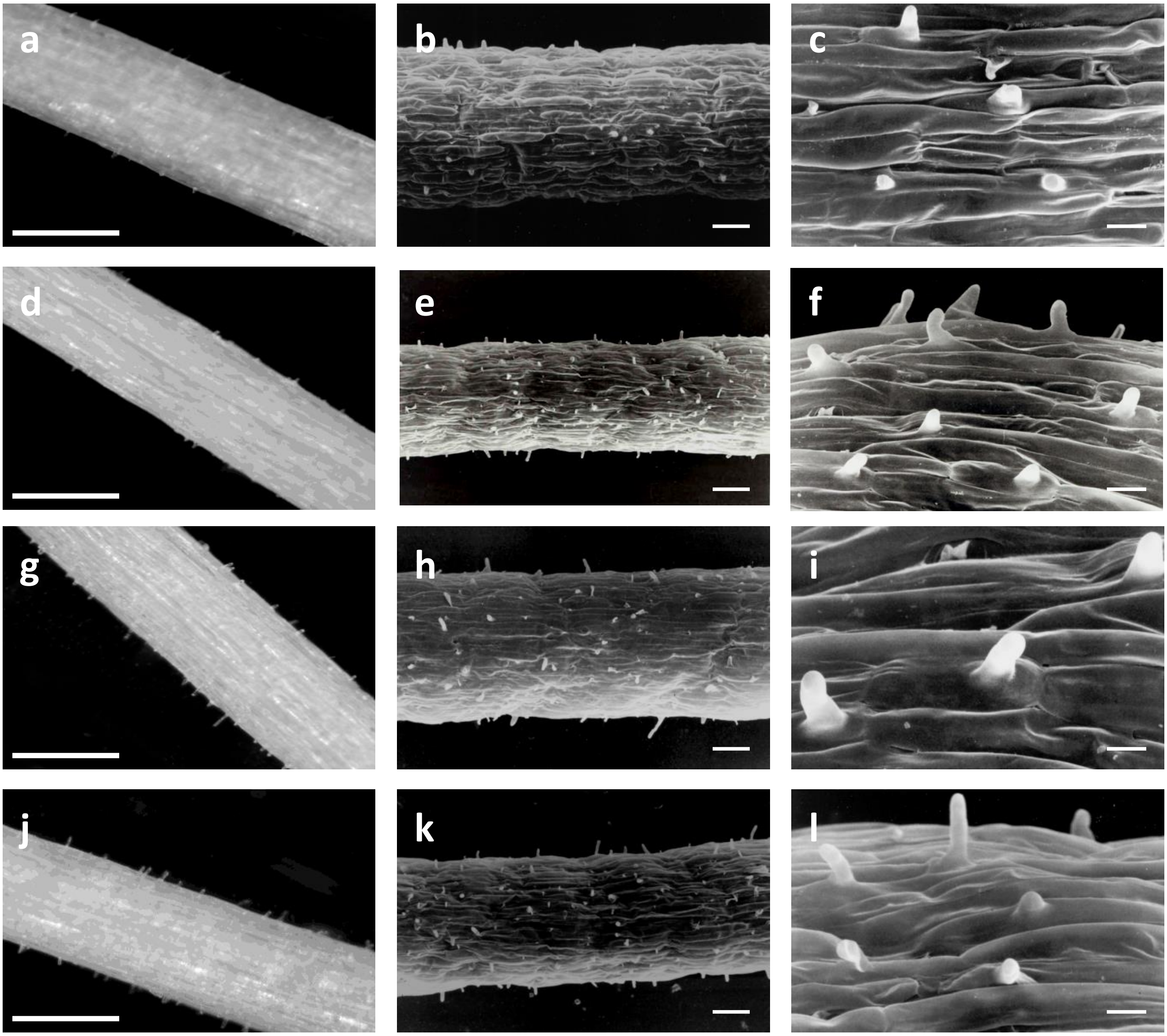

ESM9

a,b,c - *rhp1.a;* d,e,f - *rhp1.b;* g,h,i - *rhp1.c;* j,k,l - *rhp1.d*

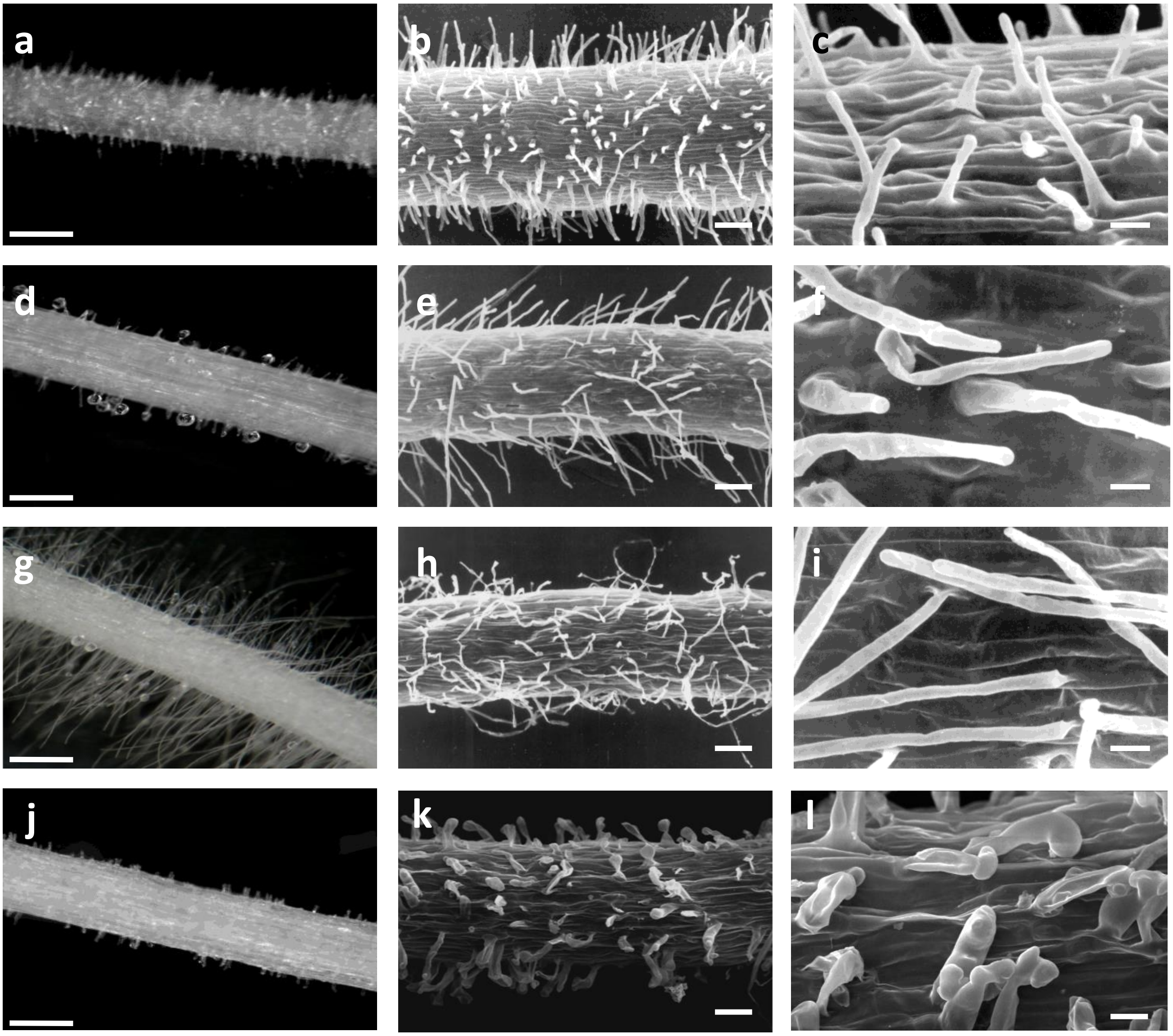

ESM10

a,b,c - *rhs1.a;* d,e,f - *rhs2.a;* g,h,i - *rhs3.a;* j,k,l - *rhs4.a*

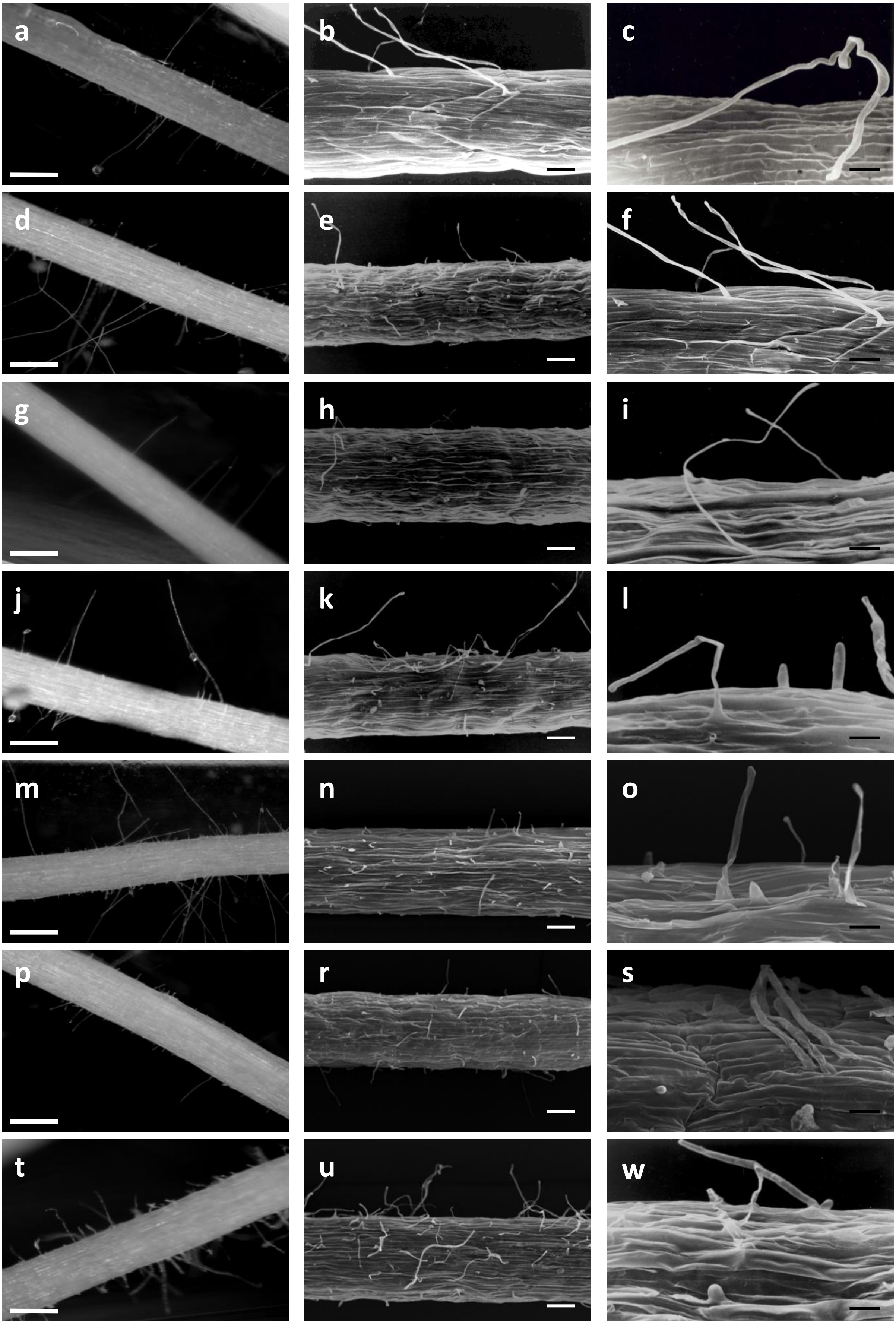

ESM11

a,b,c - *rhi1.a;* d,e,f - *rhi2.a;* g,h,i -*rhi2.b;* j,k,l - *rhi2.c;* m,n,o - *rhi2.d;* p,r,s - *rhi3.a;* t,u,w - *rhi3.b*
